# Estimating the Global, Regional, and National Economic Costs of COVID-19 Vaccination During the COVID-19 Pandemic

**DOI:** 10.3390/vaccines13111153

**Published:** 2025-11-11

**Authors:** Yansheng Chen, Haonan Zhang, Chaofan Wang, Hai Fang

**Affiliations:** 1 School of Public Health, Peking University, Beijing 100191, China; cys.alan@stu.pku.edu.cn (Y.C.); zhn@pku.edu.cn (H.Z.); chaofan@stu.pku.edu.cn (C.W.); 2 China Center for Health Development Studies, Peking University, Beijing 100191, China; 3 Peking University Health Science Center-Chinese Center for Disease Control and Prevention Joint Center for Vaccine Economics, Beijing 100191, China

**Keywords:** COVID-19 vaccination, vaccination cost, global, cost disparity, health Policy

## Abstract

**Background:** The COVID-19 pandemic led to an unprecedented global health and economic crisis, and vaccination emerged as a critical intervention to control the spread of the virus and mitigate its impact on health systems and economies. Despite the rapid development and deployment of vaccines, the financial commitments required for these vaccination programs are substantial, necessitating a comprehensive understanding of the associated costs to inform future public health strategies and resource allocation. **Method:** This analysis estimates the global, regional, and national economic costs of COVID-19 vaccination across 234 countries and regions in the period 2020–2023, consisting of vaccine procurement costs and administration costs. **Result:** As of 31 December 2023, the global costs of COVID-19 vaccination programs were estimated at USD 246.2 billion, with vaccine procurement accounting for approximately USD 140.2 billion and administration costs totaling USD 96.4 billion. Globally, a cumulative total of 136.9 billion doses of COVID-19 vaccines had been administered. Factoring in an estimated wastage rate of 10%, it is projected that approximately 150.6 billion doses were used. On a global scale, the average number of vaccine doses administered per capita was estimated at 1.73. The mean cost per capita was USD 17.70 (95% CI: USD 15.84–19.56) for vaccine procurement and USD 12.16 (95% CI: USD 10.29–14.02) for administration, resulting in a total average cost of USD 29.85 (95% CI: USD 26.33–33.37) per capita. Significant disparities in costs were observed across income groups and regions. High-income countries incurred a notably higher average cost per capita of USD 76.90 (95% CI: USD 72.38–81.41) in contrast to low-income countries, where the per capita cost was USD 7.20 (95% CI: USD 5.38–9.02). For middle-income countries, the average per capita costs were USD 15.02 (95% CI: USD 10.64–19.40) in lower-middle-income countries and USD 28.21 (95% CI: USD 23.60–32.83) in upper-middle-income countries. Regionally, the Americas (AMR) reported the highest total cost at USD 70.8 billion, with an average per capita cost of USD 65.23 (95% CI: USD 56.18–74.28). The Western Pacific Region (WPR) followed with a total cost of USD 63.9 billion and an average per capita cost of USD 31.93 (95% CI: USD 20.35–43.51). Conversely, the African Region (AFR) had the lowest total spending at USD 10.8 billion and a per capita cost of USD 8.85 (95% CI: USD 5.34–12.37), reflecting both lower vaccine procurement and administration costs. The European Region (EUR) recorded a high average per capita cost of USD 53.36 (95% CI: USD 46.79–59.94), with procurement costs at USD 31.28 (95% CI: USD 27.41–35.14) and administration costs of USD 22.09 (95% CI: USD 19.31–24.87). **Conclusions:** The global rollout of COVID-19 vaccination revealed substantial variation in cost structures across income groups. Procurement costs imposed greater burdens on low- and lower-middle-income countries, whereas delivery and administration costs dominated in higher-income settings. These disparities highlight persistent fiscal inequities and emphasize the need for stronger international coordination and cost transparency to enhance equity, efficiency, and preparedness in future vaccination efforts.

## 1. Introduction

The Coronavirus Disease 2019 (COVID-19) pandemic caused an unparalleled global health crisis, profoundly impacting global health systems and economies. With more than 770 million confirmed cases and more than 7 million deaths worldwide by January 2024 [1], the pandemic has underscored the critical importance of robust public health responses. Confronted with the COVID-19 pandemic, governments and international organizations have undertaken a wide range of public health interventions. Among these, vaccination has emerged as the most effective measure to curb the spread of Severe Acute Respiratory Syndrome Coronavirus 2 (SARS-CoV-2), the virus responsible for COVID-19 [2]. Vaccination has been effective in reducing transmission; significantly reducing morbidity, severe illness, and mortality; alleviating the burden on the healthcare system; preventing labor losses; and even saving costs [3].

The global COVID-19 vaccination campaign was implemented in phases. Initially, vaccine development was accelerated by scientific collaborations, and clinical trials of various vaccines were launched intensively. The vaccination began in late 2020, when some vaccines received emergency use authorizations (EUAs) and were approved for vaccination in adults. By 2021, a total of 16 vaccines had been granted EUA globally, and over 33 vaccines were in Phase III trials and nearly 130 were in earlier development [4]. Vaccinations at this time were prioritized for healthcare workers, elderly people, and individuals with comorbidities, and were later expanded to a broader adult population to achieve herd immunity and reduce the burden of disease. Children and adolescents were included after the careful evaluation of safety and efficacy, with vaccination for younger groups beginning in early 2022 in some regions [5]. The vaccination strategy has been adapted since then as COVID-19 outbreaks have progressed, incorporating booster doses and revaccination, with the aim of providing more comprehensive and reliable protection.

While the rapid deployment of COVID-19 vaccines represents a remarkable achievement in global health collaboration, these efforts come with substantial financial commitments. The economic costs associated with these vaccination programs encompass a wide range of expenditures, including vaccine procurement, cold chain logistics, healthcare workforce mobilization, public communication, and the infrastructure required to support mass vaccination efforts. Estimating the cost of COVID-19 vaccination is critical to assessing the investment in combating the outbreaks and responding to future pandemics. The evaluation of vaccination costs across regions and countries would help to optimize the allocation of limited resources to ensure that the maximum health benefit is achieved with available funds. Economic evidence could also be provided to ensure sustained funding and support for vaccination programs, particularly in low- and middle-income countries where budget constraints are more pronounced. Moreover, a comprehensive understanding of vaccination costs could inform future pandemic preparedness and response strategies, ensuring that health systems worldwide are better equipped to manage similar crises in the future.

Several studies have been conducted to evaluate the costs associated with COVID-19 vaccination in specific countries, providing insights into specific components such as vaccine procurement, distribution logistics, cold chain requirements, and administrative expenses. For instance, a study in Kenya estimated the incremental cost of COVID-19 vaccine delivery from a health system perspective. The results of the study showed that, at 30% and 100% coverage levels, the financial cost of two doses of vaccine per capita ranged from USD 2.89 to USD 13.09, and the economic cost for all strategies was USD 17.34. In addition to procurement, the main financial cost drivers were supply chain costs, and the main economic cost drivers were advocacy, communication, and social mobilization costs [6]. By exploring the cost factors associated with vaccine delivery, this study has underlined the possible logistical complexity of global vaccination campaigns. Another study assessed the cost of COVID-19 vaccination in Vietnam in 2021 from a payer perspective, and found that the economic cost for a dose of COVID-19 vaccine was USD 1.73, with the highest cost of vaccination in urban areas, followed by peri-urban areas and remote areas [7]. This indicated that regional differences might exist in the cost of vaccination due to economic conditions, accessibility of healthcare, etc. Despite these contributions, there remains a significant gap in the literature concerning a comprehensive, multi-country analysis of the total costs of COVID-19 vaccination programs with a global perspective. Existing research often focuses on individual countries or regions, which limits comparisons of findings across countries and regions.

To address these gaps, this study involves an extensive and detailed analysis of both direct and indirect costs associated with COVID-19 vaccination programs across 234 countries and regions. Specifically, this study aims to quantify the costs of vaccination among countries and regions with varying economic status and healthcare infrastructure. By systematically analyzing cost components such as vaccine procurement, distribution logistics, healthcare workforce mobilization, and public outreach initiatives, this study aims to provide a comprehensive assessment of the financial investments required by countries and regions in their efforts to achieve widespread immunization. This analysis of the global cost of COVID-19 vaccination is intended to inform policymakers and global health stakeholders, facilitating the optimization of resource allocation and the development of more equitable and sustainable vaccination strategies in protecting against future pandemics.

## 2. Method

### 2.1. Research Design

This study utilizes a cost analysis methodology to evaluate the global economic impact of COVID-19 vaccination programs from the perspective of healthcare systems until 31 December 2023. The primary objective is to quantify the costs associated with mass vaccination campaigns across 234 countries and regions to assess the resource costs associated with immunization in COVID-19 epidemics in different countries. The population included in the study consisted of all individuals who met the requirements for COVID-19 vaccination, including vaccinees of all ages. Given the short time span of the study, no discount rate was applied. In calculating the total costs at the national level, a wastage rate of 10% was used in this study, and the number of doses after accounting for wastage was taken as the final consumption in each country, following the cost analysis by the World Health Organization (WHO) for 92 countries [8] in 2021.

### 2.2. Cost Analysis

The cost analysis incorporates expenditures for the entire process from vaccine acquisition to injection. The costs associated with the launch and execution of the COVID-19 vaccination initiative were categorized into two principal components [9]: vaccine procurement and administrative costs. Vaccine procurement refers primarily to the cost of purchasing vaccines and related supplies, while administrative costs refer to costs associated with the vaccination program other than vaccine procurement. Administrative costs include expenditures on personnel, i.e., salaries and allowances paid to health workers and related personnel for vaccination; costs associated with the vaccine supply chain, i.e., capital costs for the procurement of cold chain equipment, waste management and control equipment, vehicles for last mile distribution, and renovations of vaccine warehouses; training expenses, i.e., costs incurred in training relevant personnel; advocacy, communication, and social mobilization efforts, i.e., expenditures to mobilize the general public for vaccination and to increase vaccination coverage; and data management, monitoring, and supervision costs.

To obtain the country-level cost of COVID-19 vaccination, the per-dose costs of procurement and administration were multiplied by the number of vaccine doses administered in each country, yielding the corresponding total cost of procurement and administration. For vaccine procurement costs, a weighted average method was adopted in order to estimate the average procurement price per vaccine dose in each country, using the prices of different producers and the quantity of vaccine supplied by each producer in different countries. The specific estimation process is presented in Supplementary Material S1. For vaccine administration costs, costs per dose of vaccine administered in each country were obtained directly from existing publicly available databases and literature. For countries and regions where specific information was not available, a dual-restriction method based on income level and region was applied to estimate the administration costs. In cases where no regional data was available, the average costs of administration in countries of the same income level were used as the administrative costs for these regions. The average here represents an averaging of management costs obtained from databases and the literature and excludes the costs for countries obtained using the dual-restriction estimates. The total procurement costs and total administrative costs for each country were estimated and then divided by the number of vaccinated individuals in each country to obtain the cost per capita. Moreover, to enable macro-level comparisons of COVID-19 vaccination inputs in countries across different regions and economic levels, the aggregate costs for regions or different income groups were calculated based on WHO and World Bank stratification for regions and income levels.

### 2.3. Data Source

The cost data collected for this study comprises three main components: sociodemographic characteristics, vaccine procurement cost, and vaccine administration cost. The sociodemographic characteristics information includes a list of countries and regions, the WHO regional classification, income level groupings, and population size for each country. Data acquired on vaccine procurement prices and vaccine administration costs were primarily in the form of cost per dose. The study derived data from a wide range of data sources, including existing research literature, official reports, and publicly available databases. Specific data sources are elaborated upon in the following subsections, organized by data category. The overall data sources are presented in Table A1 and the parameter values and data sources for each country are detailed in Table A2.

#### 2.3.1. Sociodemographic Characteristics

In terms of socioeconomic characteristics, national global data were obtained from official sources. The lists of countries and regions were derived from Coronavirus (COVID-19) Vaccinations of Our World in Data [1]. The population data were derived from the World Population Prospects by the United Nations [10]. The stratification of countries by income and region were based on classifications by Country and Lending Groups from the World Bank [11] and the WHO [12], respectively.

#### 2.3.2. Vaccination Dosage

The total doses administered for 206 countries or regions were collected from WHO COVID-19 dashboard data (160 countries) and the COVID-19 Vaccination dashboard of the Africa Centre for Disease Control and Prevention (Africa CDC) (46 countries). For the remaining 28 countries without official data on doses, the vaccination rate was assumed to be consistent with the global average vaccination rate provided by the WHO, i.e., 171.88 doses per 100 population. The average vaccination rate was used to estimate the total number of doses administered by multiplying it with the population of these countries.

#### 2.3.3. Vaccine Procurement Cost

The average vaccine procurement price for each country was calculated using a weighted average of the quantities supplied by different manufacturers and the corresponding vaccine prices. The prices per dose of vaccines from different manufacturers were obtained from the Market Information for Access to Vaccines (Mi4A) dataset and publicly available information, as specified in Supplementary Material S1. Data on vaccine manufacturers and the corresponding number/percentage of doses administered were obtained from Our World in Data [13], the Africa CDC [14], and the Pan American Health Organization (PAHO) [15]. Data on supply volumes by different manufacturers was available directly for 107 countries, with Our World in Data providing data for 56 countries, the Africa CDC for 46 countries, and the PAHO for 3 countries. For other countries where data on the number of doses supplied by each manufacturer were not available, estimates were made using an equal distribution among all approved manufacturers in these countries, forming a relatively conservative estimate. The specific approach and process for estimating the average price of COVID-19 vaccines for each country is illustrated in Supplementary Material S1.

#### 2.3.4. Administration Costs

For the cost per dose of vaccine administration, data for 23 low- and middle-income countries were extracted from the Immunization Delivery Cost Catalogue (IDCC), a comprehensive resource covering over 22,000 articles from 2005 to 2023. The costs were categorized by the IDCC into incremental and full costs according to whether or not the inherent costs of other vaccinations were included, and into financial and economic costs according to whether or not the value of donated goods and services was considered. The financial cost analysis covers only the actual expenditure on capital equipment without factoring in the value of any donated goods or services. The economic cost, by contrast, captures the annualized value of the capital investment as well as the contribution of donated goods and labor. According to the objectives to estimate the incremental cost of COVID-19 vaccinations borne by the health system, the incremental financial cost from the IDCC data is preferred.

However, the data on the cost of administration is lacking in some countries, and therefore a literature review was conducted to fill in the blanks in these countries. Nine databases (PubMed, Cochrane Library, Embase, Scopus, Web of Science, Econlit, CNKI, Wanfang Data, CQVIP) were searched to identify studies involving the cost analysis of COVID-19 vaccination across countries. The details of the literature review are provided in Supplementary Material S3. Data on the administrative cost of COVID-19 vaccination were obtained for 46 countries based on the methodology adopted by the IDCC for the collection of cost data. For the remaining countries where country-specific data for administration costs were not available, a dual restriction based on the income level and region subgroups as defined by the WHO was applied and estimated, as demonstrated in Supplementary Material S2.

## 3. Results

### 3.1. Global Level

By 31 December 2023, it was estimated that a total of 15.06 billion doses of COVID-19 vaccines had been administered and consumed globally, factoring in a 10% wastage rate. Within this global context, China had the most extensive vaccination campaign, with a total of 3.84 billion doses of vaccine. At the individual level, the global average number of vaccine doses administered per capita was estimated at 1.73. Yemen recorded the lowest average number of doses, with only 0.04 doses per capita, while Cuba reported the highest average at 4.07 doses per capita. The total costs of COVID-19 vaccination programs worldwide amounted to approximately USD 246.2 billion. Notably, over half of this amount—around USD 140.3 billion—was allocated for vaccine procurement, while USD 96.4 billion was dedicated to vaccine administration.

**Figure 1:**
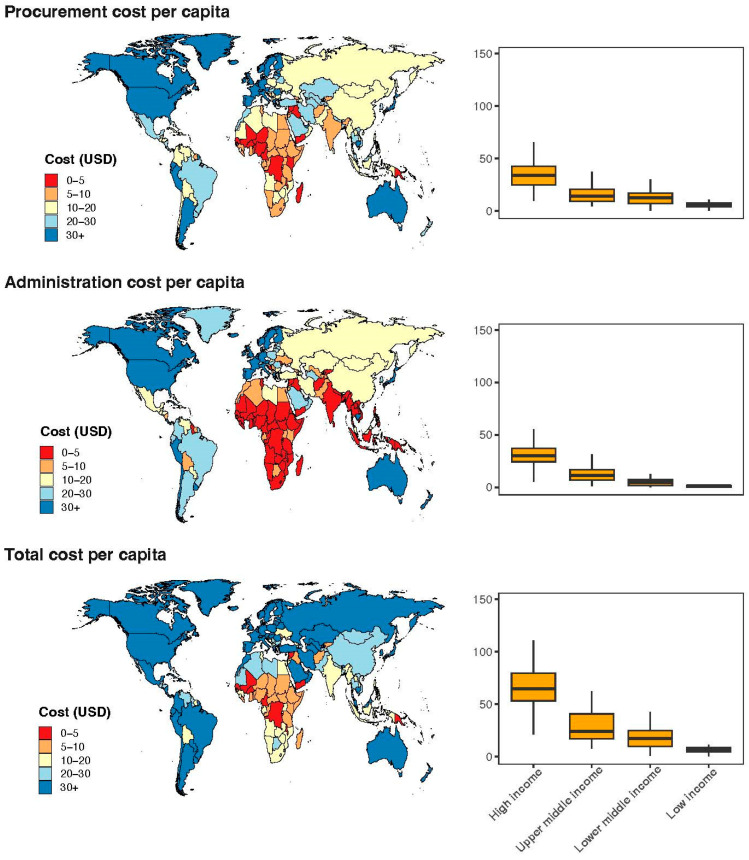
Per capita costs of COVID-19 vaccinations by countries.

**Figure 2:**
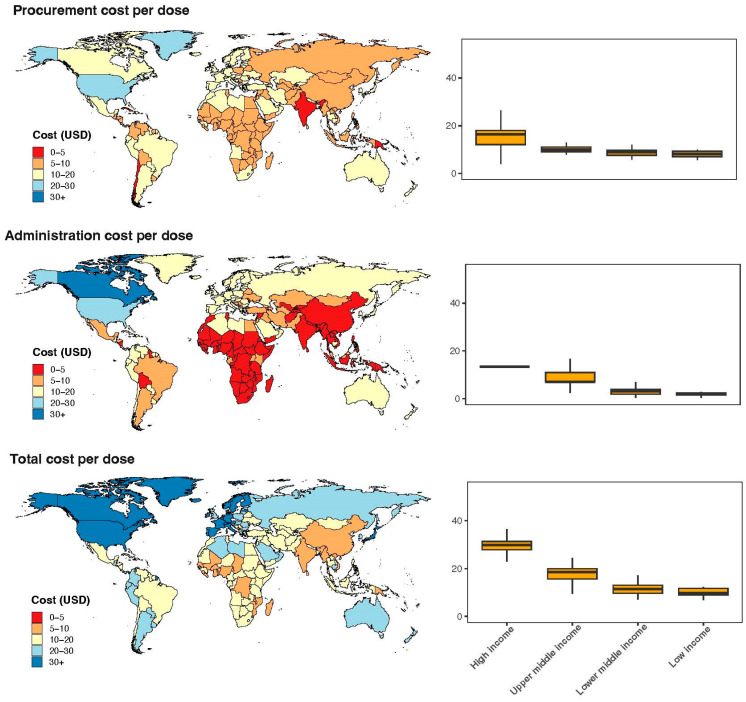
Per-dose costs of COVID-19 vaccinations by countries.

Globally, the average cost per dose of COVID-19 vaccines was estimated to be USD 11.97 (95% CI: 11.37–12.57), with an additional USD 9.38 (95% CI: 8.54–10.23) for vaccination administration costs. Collectively, these expenses constituted a total per-dose cost of USD 21.35 (95% CI: 20.04–22.66) for COVID-19 vaccines. Furthermore, at the individual level, the global average unit cost for vaccine procurement was estimated at USD 17.70 (95% CI: 15.84–19.56) per capita, with costs ranging from USD 0.32 in Papua New Guinea to USD 65.52 in Japan. The average cost of administering a vaccine was USD 12.16 (95% CI: 10.29–14.02), varying from USD 0.02 in Togo to USD 85.68 in Canada. Thus, the combined costs of procurement and administration resulted in an average total cost of USD 29.85 (95% CI: 26.33–33.37) per capita, varying from USD 0.49 in Yemen to USD 140.02 in Canada. The majority of countries in the Americas (including North and South America) and Europe had a per capita vaccination cost exceeding USD 30, whereas most African countries had a per capita vaccination cost below USD 20 (Figure 1). The cost per dose also followed the same pattern, with the Americas and Europe generally higher than Africa and Asia (Figure 2). Detailed unit cost data are presented in Table A3.

As of 2024, the COVID-19 vaccines in global use comprised four principal platforms produced by more than a dozen manufacturers: mRNA (Pfizer/BioNTech [Comirnaty], Pfizer: New York, NY, USA; BioNTech: Mainz, Germany; Moderna [Spikevax], Cambridge, MA, USA); adenoviral vector (Johnson & Johnson/Janssen [Janssen COVID-19 Vaccine], New Brunswick, NJ, USA; Oxford University/AstraZeneca [Covishield, Serum Institute of India], Cambridge, UK; Gamaleya Research Institute [Sputnik V, Sputnik Light], Moscow, Russia; CanSino Biologics [Convidecia], Tianjin, China); inactivated whole-virion (Sinovac Biotech [CoronaVac], Beijing, China; Sinopharm [BIBP/BBIBP-CorV], Beijing, China; Bharat Biotech [Covaxin], Hyderabad, India); and protein subunit/other (Novavax [NVX-CoV2373], Gaithersburg, MD, USA; Sanofi/GSK [Sanofi-GSK COVID-19 Vaccine], Sanofi: Paris, France; GSK: London, UK; SK bioscience [SKYCovione], Seongnam, Republic of Korea; Valneva [Valneva COVID-19 Vaccine], Saint-Herblain, France; Medicago [Covifenz], Quebec City, QC, Canada; Biological E [Corbevax], Hyderabad, India). mRNA products and Oxford/AstraZeneca accounted for the largest share of country-level uptake, with Johnson & Johnson also widely deployed. Sinopharm and Sinovac were extensively used, particularly across Asia, the Middle East, Africa, and Latin America, while other products represented a smaller fraction of global use. Regarding pricing, Moderna carried the highest per-dose prices in high-income settings (up to USD 26.48), with contracts elsewhere around USD 10 per dose; Pfizer/BioNTech ranged from roughly USD 7 (low-income) to USD 17.24 (high-income) per dose. Oxford/AstraZeneca (including COVAX supply) was typically priced at about USD 4 per dose. Sinopharm (BIBP/BBIBP-CorV) and Sinovac (CoronaVac) were commonly priced at approximately USD 19 and USD 14–15.48 per dose, respectively. Prices for other vaccines generally fell between USD 1.92 and USD 15 per dose (detailed information in Supplementary Materials).

From 2020 to 2024, China and the United States were the two largest producers, users, and cross-border suppliers of COVID-19 vaccines; both achieved approximately two doses per capita over this period. Their expenditure patterns diverged. In the United States, procurement and administration costs averaged USD 39.28 and USD 29.84 per dose, with administration accounting for about USD 19 billion of the USD 34 billion in total program costs. China recorded lower unit costs (USD 14.41 for procurement and USD 10.31 for administration) alongside far larger absolute dose volumes. External provision also differed in scale and composition. The United States pursued a donation-led strategy, donating 693 million doses to 117–118 countries and economies, with about 89% via COVID-19 Vaccines Global Access (COVAX) and the remainder bilaterally [16]. The US donation mix was dominated by Pfizer–BioNTech (76%), followed by Moderna (12%), Janssen (10%), and AstraZeneca (2%) [17]. China also donated a large number of doses (roughly 239–328 million), primarily Sinopharm (BBIBP-CorV; roughly 103 million delivered by end-2022) and Sinovac (CoronaVac), with smaller volumes of CanSino and Anhui Zhifei [18].

### 3.2. Cost by Income Level

Significant disparities existed in the number of vaccine doses administered across countries at varying income levels. Middle-income countries accounted for the largest share, administering 75.2% of all doses. Among them, upper-middle-income countries utilized 6.49 billion doses with 2.09 doses per capita, while lower-middle-income countries administered 1.46 doses per capita, resulting in 4.83 billion doses in total. In contrast, 85 high-income countries collectively consumed 3.23 billion doses (21.4%) with 2.13 doses per capita, and 25 low-income countries used only 440 million doses (2.9%) with only 0.60 doses per capita.

Expenditure patterns also revealed considerable differences among income levels, with 25 low-income countries allocating USD 4.9 billion, while 82 high-income countries spent a total of USD 106.0 billion on COVID-19 vaccination programs. Additionally, 50 lower-middle-income countries and 54 upper-middle-income countries spent USD 45.3 billion and USD 79.6 billion, respectively, with significant contributions from India and China, each with populations exceeding 1.4 billion.

The differences in cost per dose of vaccine were significant. On average, the cost per dose of COVID-19 vaccines was highest in high-income countries, amounting to USD 30.34 (95% CI: 28.75–31.93), followed by upper-middle-income countries at USD 18.30 (95% CI: 16.76–19.84) per dose and lower-middle-income countries at USD 12.63 (95% CI: 10.90–14.35) per dose. The lowest costs per dose were observed in low-income countries, with an average of USD 10.55 (95% CI: 9.53–11.58).

At the individual level, high-income countries reported an average cost of USD 76.90 (95% CI: 72.38–81.41) per capita, which was significantly higher than USD 7.2 (95% CI: 5.38–9.02) by low-income countries, USD 15.02 (95% CI: 10.64–19.40) by lower-middle-income countries, and USD 28.21 (95% CI: 23.60–32.83) by upper-middle-income countries. It is worth noting that due to the World Bank’s classification limitations, the total costs across these income groups did not sum to the global total, and in some cases, countries lacked a designated region. Detailed cost data are available in Table 1.

### 3.3. Cost by Region

Among the six regions, the WPR (Western Pacific Region) consumed and used the highest number of 2.42 doses per capita, totaling 5.12 billion (34.0%). The SEAR (South-East Asia Region) followed closely with 1.64 doses per capita and 3.69 billion doses (24.5%) in total, with both regions including populous countries such as China and India. The AMR (Americas Region) ranked third, using 2.32 billion doses (15.4%) with 2.05 doses per capita, followed by the EUR (European Region) with 1.95 billion doses (12.9%) and 1.90 doses per capita. The EMR (Eastern Mediterranean Region) and AFR (African Region) had the lowest figures, consuming 1.00 billion (6.7%) and 0.92 billion (6.1%) doses, with 1.18 and 0.70 doses per capita, respectively.

In terms of vaccination costs, the region with the highest expenditure was AMR, consisting of 35 countries, with total expenditures of USD 67.3 billion. This was followed by WPR, which included only 9 countries and spent USD 61.5 billion, and EUR, which included 53 countries and spent USD 49.8 billion. SEAR, with 27 countries, recorded spending of USD 29.7 billion, while EMR, with 21 countries, spent USD 16.8 billion. Although AFR included 47 countries, it had the lowest spending at only USD 10.6 billion.

In terms of expenditure, SEAR recorded the lowest cost per dose at USD 10.31 (95% CI: 9.02–11.60). Slightly above was AFR, at USD 12.01 (95% CI: 10.80–13.24) per dose. EMR had the third-lowest cost per dose, at USD 17.86 (95% CI: 15.52–20.20). The remaining three regions had costs per dose exceeding USD 20, with WPR at USD 20.78 (95% CI: 15.87–25.69) per dose, AMR at USD 22.60 (95% CI: 19.61–25.68) per dose, and EUR with the highest cost at USD 25.83 (95% CI: 24.00–27.66) per dose. Furthermore, a regional cost analysis revealed that AFR had notably lower average costs, expending USD 8.85 (95% CI: 5.34–12.37) per capita, with USD 6.62 (95% CI: 4.41–8.88) for procurement and USD 2.23 (95% CI: 0.73–3.73) for administration. AMR had a vaccine procurement cost of USD 30.89 (95% CI: 27.12–34.78) and incurred a significantly higher administration cost of USD 34.34 (95% CI: 28.53–40.14) per capita, leading to the highest cost of USD 65.23 (95% CI: 56.18–74.28) per capita. EMR experienced a total cost of USD 21.64 (95% CI: 12.31–30.96) per capita, with a procurement cost of USD 13.29 (95% CI: 8.56–18.02) but a lower administration cost of USD 8.35 (95% CI: 3.30–13.41). In contrast, European countries reported an average cost of USD 53.36 (95% CI: 46.79–59.94) per capita, with the highest procurement cost at USD 31.28 (95% CI: 27.37–35.18) and an administration cost of USD 22.09 (95% CI: 19.31–24.87). WPR and SEAR had procurement costs of USD 19.59 (95% CI: 13.27–25.91) and USD 11.08 (95% CI: 6.76–15.40) per capita, respectively, with administration costs of USD 12.34 (95% CI: 6.20–18.47) and USD 3.45 (95% CI: 1.59–5.31) per capita, resulting in total costs of USD 31.93 (95% CI: 20.35–43.51) and USD 14.53 (95% CI: 10.31–18.75) per capita. Details are available in Table 2.

## 4. Discussion

This study presents a comprehensive assessment of global COVID-19 vaccination expenditures, distinguishing vaccine procurement from delivery and administration costs across countries and income groups. Overall, the results reveal substantial heterogeneity in both the magnitude and structure of spending: procurement dominates the total costs in many low- and lower-middle-income settings, while delivery-related expenses are relatively higher in upper-middle- and high-income countries with more complex health-service systems. Such differences reflect the interaction between financing capacity, labor and infrastructure costs, and the organization of immunization services. Interpreting these variations helps to clarify how economic and system characteristics shape the fiscal burden of vaccination and provides a foundation for examining the implications for resource allocation, financing sustainability, and global equity.

In the statistics of this study, a total of 13.69 billion doses of vaccines were administered globally and 15.06 billion doses were produced and consumed after accounting for 10% vaccine wastage. This is closely aligned with the 13.64 billion doses documented by the WHO COVID-19 dashboard [1]. However, this estimate appears conservative when juxtaposed with the 2023 Global Vaccine Market Report by the WHO [19], which projects a total global consumption of 18.8 billion doses and a market value of USD 179 billion. The divergence in vaccine doses may stem from the different perspectives of estimation, as this study prioritized the number of vaccination doses reported by governments and applied a 10% wastage rate, while the WHO report incorporates market estimates from the perspective of market supply, factoring in greater wastage due to the rapid development of vaccines and suboptimal storage conditions. In addition, the difference in dose estimates contributes to the difference in investment valuations.

Regionally, considerable variation exists in the cost of COVID-19 vaccinations, with the high-income-country-dense EUR region reporting a total cost of USD 53.36 per capita, while the AFR region shows the lowest average cost of USD 8.85 per capita. Moreover, differences existed in the share of cost components. In AFR, EUR, WPR, and SEAR regions, procurement costs were higher than the administration costs, while AMR and EMR regions exhibit the opposite trend. This might be attributed to the geographic conditions that might affect transportation. In AMR and EMR, vaccines must navigate complex logistical networks to reach these regions, which increases cold chain transportation costs [20]. In addition, excessive labor costs can also increase the cost of vaccination [21]. This is especially critical for COVID-19 vaccines, as many of these require transport and storage at extremely low temperatures, exacerbating logistical challenges and costs [22].

Regarding income levels, the share of cost composition also differs. Low-income and lower-middle-income countries experience higher procurement costs compared to administrative costs. The relatively low cost of administration might arise from the lower cost of labor. Meanwhile, the comparatively large proportion of vaccine procurement costs may put significant financial pressure on these regions, especially when purchased in large quantities [23,24]. Similar results were reported in another study, which estimated that 67% of the total cost of herd immunization through vaccination was spent on vaccine procurement and 33% on vaccine delivery in low- and middle-income countries [25]. This indicates that despite lower administration costs in these areas, high procurement costs remain a significant challenge.

The high procurement costs might be due to the lack of price transparency and the strong bargaining power of vaccine manufacturers in developed countries. The study of COVID-19 vaccine pricing policies have indicated that high-income countries have managed to gain priority in vaccine supply queues by contracting directly with manufacturers, while low- and middle-income countries have struggled to secure adequate supplies due to financial constraints and limited negotiating power [26]. Although the COVAX mechanism was intended to reduce vaccine prices through pooled procurement, competitive purchasing practices in high-income countries have weakened the collective purchasing power of COVAX, further exacerbating inequalities in vaccine distribution [27]. Although low-income and lower-middle-income countries often face significant economic constraints and struggle to secure sufficient vaccine supplies [27], increasing international vaccine aid has enabled them to obtain adequate vaccines at relatively lower prices [28]. This support has helped improve vaccination rates in these countries. Additionally, enhancing vaccine production capacity within these nations or regions can contribute to long-term improvements in public health security [29]. This is particularly relevant for African countries, which rely on imports for 99% of their vaccines [30].

Conversely, high- and upper-middle-income countries exhibit stronger bargaining power in the procurement of COVID-19 vaccines. Most of the WHO-approved vaccines come from these countries, including the United States, the United Kingdom, Russia, and China, which strengthens their leverage in price negotiations [31]. Despite their negotiating strength, procurement costs in high- and upper-middle-income countries have not significantly decreased due to their exclusion from COVAX’s primary target audience. In many cases, procurement costs in high-income countries are even higher than in low-income countries that might receive donation support for vaccines. Furthermore, high- and upper-middle-income countries generally possess healthcare systems of a larger scale that incur higher operational costs while enabling effective vaccine administration [32]. In these countries and regions, labor costs and healthcare facility maintenance costs account for a substantial portion of the administration costs. For instance, another study shows that the cost of operational staffing and operational facilities and services in American clinics accounts for 84.2% of the total operational cost [33].

To illustrate how these procurement and delivery dynamics played out in practice, we compared China and the United States, the two largest providers of COVID-19 vaccines during 2020–2024. The United States took a donation-led approach: it donated approximately 693 million doses, committed USD 4.0 billion to Gavi’s COVAX Facility, and, through the U.S. International Development Finance Corporation and other tools, financed manufacturing and fill-finish capacity in partner countries. China supplied more than 2.2 billion doses across channels; within this total, free donations were about 328 million doses. Chinese manufacturers also signed advance-purchase agreements with COVAX for up to 550 million doses. These patterns reflect differences in systems and product mix. In the United States, the federal government bought large quantities for donation and, as supply improved, shifted to delivery support (cold chain, staffing, micro-planning, community outreach). The mRNA-dominated product of the US required tighter cold-chain control and more service inputs, which was consistent with higher procurement and delivery costs per dose. In China, centralized coordination and close links between government and manufacturers supported the rapid scale-up of inactivated vaccines, which had simpler cold-chain needs. External supply relied mainly on sales, with donations and multilateral procurement as complements, and later moved toward local production and fill-finish with partner countries. These strategic and product differences also shape downstream health gains and economic returns from vaccination. The public health administrative systems in both China and the United States played critical roles in the domestic distribution of COVID-19 vaccines and contributed substantially to international COVID-19 vaccination efforts.

COVID-19 vaccines have significantly enhanced global public health by effectively preventing infections, reducing the severity of illness, and lowering associated treatment costs and income loss at the individual and household levels [34]. Additionally, vaccines could reduce the prevalence of COVID-19 sequelae, thereby providing long-term protection [35,36]. Beyond the benefits at the individual level, vaccination with COVID-19 vaccines can protect socioeconomic development through macroeconomic advantages. The vaccine can avoid additional healthcare costs by preventing morbidity and mortality [37] and can further reduce productivity losses, allowing more workers to return to their jobs and enhancing production, particularly in labor-intensive sectors like manufacturing and services [38]. Without interventions like vaccination, the combined supply and demand shocks from the pandemic could cause a significant GDP decline of 5.9% to 6.5% in the short term and up to 7% in the long term [39]. Additionally, macroeconomic studies have revealed that COVID-19 vaccination can protect economic growth by fostering increases in national GDP [40,41]. Therefore, investment in COVID-19 vaccines is crucial for safeguarding both public health and global economic stability. This suggests that in future responses to new pandemics, investing in vaccines is necessary and advisable, and vaccination efforts should be advanced in a timely manner. In determining investment in vaccine development and promotion, the short- and long-term value of vaccination should be thoroughly considered to avoid potential loss of benefit due to underinvestment [42].

Although data from multiple sources were incorporated into the estimation of the global and national costs of COVID-19 vaccination, several limitations remain. First, this estimation was conducted in the absence of vaccination dose data for 28 countries/regions, specific vaccination numbers by manufacturer for 127 countries/regions, and vaccination cost data for 165 countries/regions. Consequently, this may result in potential deviations from the actual situation in the respective countries. Second, various data sources might differ in terms of data collection methods, time span, indicator definitions, and reporting formats, and this may impair the accuracy and comparability of the results of the estimation. However, this is a problem that is difficult to avoid with global-scale estimates. In addition, cost estimation based on public data and literature is difficult in terms of capturing implicit costs such as long-term management, community mobilization, and health system strengthening, and therefore may not adequately reflect the complexity of actual costs.

## 5. Conclusions

In this study, a cost analysis of COVID-19 vaccination strategies worldwide was conducted. As of 31 December 2023, a total of USD 246.2 billion was spent globally on COVID-19 vaccination, with more than half of this amount allocated to vaccine procurement, while the remaining covered various costs associated with the vaccination process. Cross-regional and cross-income comparisons reveal that, despite access to multiple products through COVAX and WHO procurement, low- and lower-middle-income countries still bear heavy procurement burdens, while upper-middle- and high-income countries incur greater delivery costs consistent with more resource-intensive systems. In contrast, upper-middle-income and high-income countries incur higher administration costs due to their more developed healthcare systems. These findings highlight the persistent inequality in fiscal capacity and cost structure across countries. Understanding where spending pressures concentrate provides an empirical basis for improving global financing coordination and for future analyses of cost-effectiveness and sustainability in pandemic preparedness. Vaccination will remain central to future respiratory pandemic responses; our estimates turn the headline “billions spent” into a practical menu of procurement and delivery reforms that can make the next campaign faster, fairer, and more affordable.

## Figures and Tables

**Table 1:** Costs by income group.

Income Group	Number of Countries/ Regions	Population (Million)	Dose (Million) *	Dose/ Capita	Costs/Dose (USD)			Costs/Capita (USD)		
					PROCUREMENT COST	Administration Cost	Total Cost	Procurement Cost	Administration Cost	Total Cost
Low income	25	677.66	449.84	0.60	8.30 [7.59–9.02]	2.25 [1.56–2.94]	10.55 [9.53–11.58]	5.55 [4.15–6.96]	1.65 [1.02–2.27]	7.20 [5.38–9.02]
Lower middle income	50	3013.04	4831.64	1.46	8.83 [7.98–9.67]	3.81 [2.65–4.96]	12.63 [10.9–14.35]	10.80 [8.18–13.41]	4.22 [2.02–6.42]	15.02 [10.64–19.40]
Upper middle income	54	2820.43	6499.15	2.09	10.06 [9.36–10.77]	8.24 [7.1–9.37]	18.30 [16.76–19.84]	17.22 [14.88–19.56]	10.99 [8.26–13.71]	28.21 [23.60–32.83]
High income	82	1378.54	3230.59	2.13	15.61 [14.73–16.5]	14.73 [13.59–15.87]	30.34 [28.75–31.93]	39.86 [37.28–42.45]	37.03 [34.39–39.68]	76.90 [72.38–81.41]
Unclassified	23	36.88	52.30	1.29	8.46 [6.19–10.73]	10.36 [8.44–12.28]	18.82 [15.25–22.39]	12.00 [6.41–17.59]	13.35 [9.29–17.41]	25.35 [16.36–34.35]
World	234	7926.55	15,063.52	1.73	11.97 [11.37–12.57]	9.38 [8.54–10.23]	21.35 [20.04–22.66]	17.70 [15.84–19.55]	12.16 [10.29–14.02]	29.85 [26.33–33.37]

* 10% wastage is included.

**Table 2:** Costs by region.

Region *	Number of Countries/ Regions	Population (Million)	Dose (Million) **	Dose/ Capita	Costs/Dose (USD)			Costs/Capita (USD)		
					Procurement Cost	Administration Cost	Total Cost	Procurement Cost	Administration Cost	Total Cost
AFR	47	1191.65	918.62	0.70	8.84 [8.19–9.51]	3.16 [2.39–3.96]	12.01 [10.8–13.24]	6.62 [4.43–8.81]	2.23 [0.73–3.73]	8.85 [5.34–12.37]
AMR	35	1032.01	2322.87	2.05	9.92 [8.84–11.04]	12.67 [10.44–14.98]	22.60 [19.61–25.68]	30.89 [27.17–34.62]	34.34 [28.53–40.14]	65.23 [56.18–74.28]
EMR	21	774.67	1007.84	1.18	10.57 [9.76–11.38]	7.29 [5.36–9.22]	17.86 [15.52–20.2]	13.29 [8.67–17.90]	8.35 [3.30–13.41]	21.64 [12.31–30.96]
EUR	53	933.96	1952.61	1.90	14.82 [13.86–15.79]	11.00 [10.05–11.96]	25.83 [24–27.66]	31.28 [27.41–35.14]	22.09 [19.31–24.87]	53.36 [46.79–59.94]
WPR	27	1927.28	5121.16	2.42	10.84 [8.57–13.10]	9.94 [6.93–12.96]	20.78 [15.87–25.69]	19.59 [13.39–25.79]	12.34 [6.20–18.47]	31.93 [20.35–43.51]
SEAR	9	2043.42	3694.28	1.64	8.99 [7.31–10.66]	1.35 [0.53–2.17]	10.31 [9.02–11.6]	11.08 [7.01–15.16]	3.45 [1.59–5.31]	14.53 [10.31–18.75]
Unclassified	42	23.56	46.13	1.78	12.56 [11.01–14.11]	8.04 [6.14–9.94]	20.60 [17.60–23.60]	24.60 [21.13–28.07]	14.31 [10.53–18.08]	38.91 [32.33–45.49]
World	234	7926.55	15,063.52	1.73	11.97 [11.37–12.57]	9.38 [8.54–10.23]	21.35 [20.04–22.66]	17.70 [15.84–19.55]	12.16 [10.29–14.02]	29.85 [26.33–33.37]

* Full names for different regions are African Region (AFR), the Region of the Americas (AMR), the Eastern Mediterranean Region (EMR), the European Region (EUR), the South-East Asia Region (SEAR), and the Western Pacific Region (WPR). ** 10% wastage is included.

## Data Availability

The data presented in this study are available on request from the corresponding author.

## Supplementary Materials

The following supporting information can be downloaded at: https://www.mdpi.com/article/10.3390/vaccines13111153/s1, Table S1. Vaccine Price Input. Table S2. Country-Level Vaccine Doses by Manufacturer. Figure S1. Flow Diagram for Literature Review. Table S3. Number of Records for 9 Databases. References [43,44,45,46,47,48,49,50,51,52,53,54] are cited in the appendix tables. References [55,56,57,58,59,60,61,62,63,64,65,66,67,68,69,70,71,72,73,74] are cited in the Supplementary Materials.

## Appendix A

**Table A1:** Data sources.

Category	Source
Total vaccine Doses	WHO, Africa CDC
Vaccine Doses/Percentage from Various Manufacturers	Our World in Data, Africa CDC, PAHO
Vaccine Prices from Various Manufacturers	Market Information for Access to Vaccines (Mi4A), Public Market Information
Administration Cost	Peer-reviewed Literature, IDCC

**Table A2:** Country-level parameter input.

Country/ Region	Population (Million)	Doses (Million)		Procurement Costs (USD)		Administration Costs (USD)	
		Value	Source *	Value	Source **	Value	Source
Afghanistan	41.13	22.96	WHO	9.05	Real Distribution	2.03	Calculation
Albania	2.84	3.09	WHO	12.78	Equal Distribution	7.08	Calculation
Algeria	44.90	34.05	Africa CDC	12.90	Real Distribution	11.49	Liu, Yang et al. [34]
American Samoa	0.04	0.08	Calculation	20.46	Equal Distribution	28.40	Calculation
Andorra	0.08	0.16	WHO	15.91	Equal Distribution	13.32	Calculation
Angola	35.59	38.43	Africa CDC	11.19	Real Distribution	3.26	Liu, Yang et al. [34]
Anguilla	0.02	0.02	WHO	10.62	Equal Distribution	13.32	Calculation
Antigua and Barbuda	0.09	0.14	WHO	10.02	Equal Distribution	19.05	Calculation
Argentina	45.51	116.72	WHO	11.31	Real Distribution	8.98	Augustovski, Federico et al. [43]
Armenia	2.78	2.26	WHO	10.28	Equal Distribution	7.08	Calculation
Aruba	0.11	0.17	WHO	17.24	Equal Distribution	13.32	Calculation
Australia	26.18	254.80	WHO	13.80	Equal Distribution	13.32	Calculation
Austria	8.94	20.47	WHO	16.80	Real Distribution	13.32	Calculation
Azerbaijan	10.36	13.86	WHO	10.45	Equal Distribution	7.08	Calculation
Bahamas	0.41	0.37	WHO	10.03	Equal Distribution	19.05	Calculation
Bahrain	1.47	3.48	WHO	11.64	Equal Distribution	13.32	Calculation
Bangladesh	171.19	361.67	WHO	12.04	Real Distribution	0.34	IDCC
Barbados	0.28	0.38	WHO	10.02	Equal Distribution	19.05	Calculation
Belarus	9.53	22.51	WHO	10.00	Equal Distribution	7.08	Calculation
Belgium	11.66	29.62	WHO	17.21	Real Distribution	13.32	Calculation
Belize	0.41	0.51	WHO	8.46	Equal Distribution	10.92	Calculation
Benin	13.35	6.41	Africa CDC	8.73	Real Distribution	0.79	IDCC
Bermuda	0.06	0.13	WHO	10.62	Equal Distribution	13.32	Calculation
Bhutan	0.78	2.01	WHO	7.69	Equal Distribution	3.90	IDCC
Bolivia	12.22	14.69	WHO	7.65	Equal Distribution	3.92	Calculation
Bonaire Sint Eustatius and Saba	0.03	0.04	WHO	18.96	Equal Distribution	13.32	Calculation
Bosnia and Herzegovina	3.23	1.92	WHO	10.45	Equal Distribution	7.08	Calculation
Botswana	2.63	4.01	Africa CDC	9.80	Real Distribution	4.59	Liu, Yang et al. [34]
Brazil	215.31	486.44	WHO	10.29	Real Distribution	8.54	Augustovski, Federico et al. [43]
British Virgin Islands	0.03	0.04	WHO	15.94	Equal Distribution	13.32	Calculation
Brunei	0.45	1.29	WHO	13.31	Equal Distribution	13.32	Calculation
Bulgaria	6.78	4.72	WHO	15.92	Real Distribution	13.32	Calculation
Burkina Faso	22.67	10.23	Africa CDC	8.05	Real Distribution	0.73	IDCC
Burundi	12.89	0.80	Africa CDC	14.67	Real Distribution	2.30	Calculation
Cambodia	16.77	47.83	WHO	13.50	Real Distribution	11.67	IDCC
Cameroon	27.91	8.24	Africa CDC	8.83	Real Distribution	1.56	Liu, Yang et al. [34]
Canada	38.45	96.90	WHO	19.64	Real Distribution	34.00	Tuite, Ashleigh R. et al. [44]
Cape Verde	0.59	1.09	Africa CDC	5.88	Real Distribution	1.15	Calculation
Cayman Islands	0.07	0.15	WHO	17.24	Equal Distribution	13.32	Calculation
Central African Republic	5.58	3.54	Africa CDC	7.78	Real Distribution	2.30	Calculation
Chad	17.72	12.15	Africa CDC	7.67	Real Distribution	2.30	Calculation
Chile	19.60	62.69	WHO	4.09	Real Distribution	11.46	Augustovski, Federico et al. [43]
China	1425.89	3492.33	WHO	5.35	Official information [45]	4.21	Official information [46]
Colombia	51.87	90.93	WHO	9.86	Equal Distribution	14.07	Augustovski, Federico et al. [43]
Comoros	0.84	2.42	Africa CDC	8.75	Real Distribution	2.61	Calculation
Congo	5.97	3.46	Africa CDC	10.02	Real Distribution	2.92	Liu, Yang et al. [34]
Cook Islands	0.02	0.04	WHO	17.24	Equal Distribution	13.32	Calculation
Costa Rica	5.18	13.58	WHO	8.77	Real Distribution	10.95	Augustovski, Federico et al. [43]
Cote d’Ivoire	28.16	33.64	Africa CDC	9.80	Real Distribution	0.45	IDCC
Croatia	4.03	5.42	WHO	16.43	Real Distribution	13.32	Calculation
Cuba	11.21	45.62	WHO	3.00	Equal Distribution	10.92	Calculation
Curacao	0.19	0.26	WHO	15.91	Equal Distribution	13.32	Calculation
Cyprus	0.90	1.80	WHO	16.24	Real Distribution	13.32	Calculation
Czechia	10.49	19.03	WHO	17.23	Real Distribution	13.32	Calculation
Democratic Republic of Congo	99.01	36.83	Africa CDC	6.88	Real Distribution	2.53	IDCC
Denmark	5.88	14.96	WHO	18.22	Real Distribution	13.32	Calculation
Djibouti	1.12	2.22	Africa CDC	13.89	Real Distribution	5.51	Calculation
Dominica	0.07	0.07	WHO	9.08	Equal Distribution	10.92	Calculation
Dominican Republic	11.23	16.43	WHO	10.45	Equal Distribution	10.92	Calculation
Ecuador	18.00	39.56	WHO	12.65	Real Distribution	10.92	Calculation
Egypt	110.99	112.67	WHO	8.38	Real Distribution	8.79	Liu, Yang et al. [34]
El Salvador	6.34	11.29	WHO	10.45	Equal Distribution	10.92	Calculation
Equatorial Guinea	1.67	1.22	Africa CDC	15.23	Real Distribution	5.90	Calculation
Eritrea	3.68	6.33	Calculation	6.63	Real Distribution	2.30	Calculation
Estonia	1.33	2.20	WHO	16.53	Real Distribution	13.32	Calculation
Eswatini	1.20	0.91	Africa CDC	5.91	Real Distribution	3.97	Liu, Yang et al. [34]
Ethiopia	123.38	81.10	Africa CDC	9.82	Real Distribution	2.22	Liu, Yang et al. [34]
Faeroe Islands	0.05	0.10	WHO	18.22	Equal Distribution	13.32	Calculation
Falkland Islands	0.00	0.00	WHO	15.94	Equal Distribution	13.32	Calculation
Fiji	0.93	1.56	WHO	8.77	Equal Distribution	7.08	Calculation
Finland	5.54	13.33	WHO	18.15	Real Distribution	13.32	Calculation
France	67.81	154.49	WHO	17.95	Real Distribution	13.32	Calculation
French Guiana	0.30	0.52	Calculation	17.95	Equal Distribution	13.32	Calculation
French Polynesia	0.31	0.50	WHO	17.95	Equal Distribution	13.32	Calculation
Gabon	2.39	2.20	Africa CDC	7.89	Real Distribution	5.90	Calculation
Gambia	2.71	1.81	Africa CDC	7.10	Real Distribution	2.30	Calculation
Georgia	3.74	2.93	WHO	10.45	Equal Distribution	7.08	Calculation
Germany	83.37	192.22	WHO	17.72	Real Distribution	13.32	Joshi, K. et al. [47]
Ghana	33.48	32.35	Africa CDC	5.90	Real Distribution	3.13	Nonvignon, J. et al. [48]
Gibraltar	0.03	0.13	WHO	10.62	Equal Distribution	13.32	Calculation
Greece	10.38	22.30	WHO	14.14	Equal Distribution	13.32	Calculation
Greenland	0.06	0.08	WHO	26.48	Equal Distribution	13.32	Calculation
Grenada	0.13	0.09	WHO	8.46	Equal Distribution	10.92	Calculation
Guadeloupe	0.40	0.68	Calculation	17.95	Equal Distribution	13.32	Calculation
Guam	0.17	0.30	Calculation	20.46	Equal Distribution	28.40	Calculation
Guatemala	17.84	20.36	WHO	9.17	Real Distribution	10.92	Calculation
Guernsey	0.06	0.18	WHO	15.94	Equal Distribution	13.32	Calculation
Guinea	13.86	12.05	WHO	10.06	Real Distribution	2.61	Calculation
Guinea-Bissau	2.11	1.76	Africa CDC	6.17	Real Distribution	2.30	Calculation
Guyana	0.81	0.96	WHO	13.67	Equal Distribution	2.34	IDCC
Haiti	11.59	0.68	WHO	8.68	Real Distribution	0.92	IDCC
Honduras	10.43	17.07	WHO	6.99	Real Distribution	6.93	IDCC
Hong Kong, China	7.49	20.92	WHO	9.88	Real Distribution	1.68	Jiang, Yawen et al. [49]
Hungary	9.97	16.70	WHO	16.12	Real Distribution	13.32	Calculation
Iceland	0.37	0.81	WHO	17.95	Real Distribution	13.32	Calculation
India	1417.17	2206.75	WHO	3.89	Real Distribution	2.86	IDCC
Indonesia	275.50	448.19	WHO	9.88	Equal Distribution	0.60	Jiang, Yawen et al. [49]
Iran	88.55	155.46	WHO	10.70	Equal Distribution	8.17	Calculation
Iraq	44.50	19.56	WHO	9.66	Equal Distribution	5.93	IDCC
Ireland	5.02	13.05	WHO	17.11	Real Distribution	13.32	Calculation
Isle of Man	0.08	0.19	WHO	15.94	Equal Distribution	13.32	Calculation
Israel	9.45	18.65	WHO	17.24	Equal Distribution	13.32	Calculation
Italy	59.04	144.61	WHO	18.23	Real Distribution	13.32	Nurchis, M. et al. [50]
Jamaica	2.83	1.53	WHO	8.46	Equal Distribution	10.92	Calculation
Japan	123.95	383.75	WHO	19.24	Real Distribution	13.32	Nagano, M et al. [51]
Jersey	0.11	0.27	WHO	15.94	Equal Distribution	13.32	Calculation
Jordan	11.29	10.06	WHO	9.66	Equal Distribution	5.51	Calculation
Kazakhstan	19.40	38.36	WHO	11.95	Equal Distribution	7.08	Calculation
Kenya	54.03	32.27	Africa CDC	5.88	Real Distribution	8.06	Orangi, S. et al. [6]
Kiribati	0.13	0.22	WHO	4.00	Equal Distribution	3.90	Calculation
Kosovo	1.78	1.84	WHO	8.16	Equal Distribution	7.08	Calculation
Kuwait	4.27	8.26	WHO	11.64	Equal Distribution	13.32	Calculation
Kyrgyzstan	6.63	3.66	WHO	11.48	Equal Distribution	3.05	Calculation
Laos	7.53	11.11	WHO	9.40	Equal Distribution	1.81	IDCC
Latvia	1.85	2.90	WHO	17.47	Real Distribution	13.32	Calculation
Lebanon	5.49	5.81	WHO	9.66	Equal Distribution	5.51	Calculation
Lesotho	2.31	3.43	Africa CDC	7.56	Real Distribution	2.61	Calculation
Liberia	5.30	5.35	Africa CDC	7.14	Real Distribution	1.62	Liu, Yang et al. [34]
Libya	6.81	7.11	Africa CDC	11.70	Real Distribution	10.41	Liu, Yang et al. [34]
Liechtenstein	0.04	0.07	WHO	23.50	Real Distribution	13.32	Calculation
Lithuania	2.75	4.60	WHO	15.79	Real Distribution	13.32	Calculation
Luxembourg	0.65	1.37	WHO	18.39	Real Distribution	13.32	Calculation
Madagascar	29.61	15.66	Africa CDC	7.08	Real Distribution	2.36	Liu, Yang et al. [34]
Malawi	20.41	10.93	Africa CDC	6.58	Real Distribution	1.47	Liu, Yang et al. [34]
Malaysia	33.94	72.65	WHO	11.36	Equal Distribution	7.08	Calculation
Maldives	0.52	0.95	WHO	9.90	Equal Distribution	0.60	Calculation
Mali	22.59	8.44	Africa CDC	5.57	Real Distribution	1.36	IDCC
Malta	0.53	1.41	WHO	16.69	Real Distribution	13.32	Calculation
Marshall Islands	0.04	0.07	Calculation	20.46	Equal Distribution	28.40	Calculation
Martinique	0.37	0.63	Calculation	17.95	Equal Distribution	13.32	Calculation
Mauritania	4.74	7.64	Africa CDC	8.83	Real Distribution	2.61	Calculation
Mauritius	1.30	3.39	Africa CDC	12.94	Real Distribution	5.90	Calculation
Mayotte	0.33	0.56	Calculation	17.95	Equal Distribution	13.32	Calculation
Mexico	127.50	223.16	WHO	10.61	Equal Distribution	6.36	Augustovski, Federico et al. [43]
Micronesia (country)	0.11	0.20	Calculation	20.46	Equal Distribution	28.40	Calculation
Moldova	3.27	2.29	WHO	9.90	Equal Distribution	2.49	IDCC
Monaco	0.04	0.06	Calculation	17.24	Equal Distribution	13.32	Calculation
Mongolia	3.40	5.49	WHO	9.08	Equal Distribution	7.08	Calculation
Montenegro	0.63	0.68	WHO	9.08	Equal Distribution	7.08	Calculation
Montserrat	0.00	0.00	WHO	4.00	Equal Distribution	13.32	Calculation
Morocco	37.46	60.80	Africa CDC	15.79	Real Distribution	4.07	Liu, Yang et al. [34]
Mozambique	32.97	48.95	Africa CDC	8.22	Real Distribution	1.38	Liu, Yang et al. [34]
Myanmar	54.18	93.48	WHO	7.00	Equal Distribution	1.73	Calculation
Namibia	2.57	2.09	Africa CDC	9.93	Real Distribution	4.48	Liu, Yang et al. [34]
Nauru	0.01	0.03	WHO	4.00	Equal Distribution	13.32	Calculation
Nepal	30.55	62.63	WHO	11.71	Real Distribution	1.14	IDCC
Netherlands	17.56	39.76	WHO	18.96	Real Distribution	13.32	Calculation
New Caledonia	0.29	0.48	WHO	17.24	Equal Distribution	13.32	Calculation
New Zealand	5.19	12.35	WHO	10.62	Equal Distribution	13.32	Calculation
Nicaragua	6.95	15.51	WHO	8.74	Equal Distribution	3.92	Calculation
Niger	26.21	11.30	Africa CDC	8.32	Real Distribution	4.05	IDCC
Nigeria	218.54	133.05	WHO	6.81	Real Distribution	2.64	Liu, Yang et al. [34]
Niue	0.00	0.00	WHO	17.24	Equal Distribution	13.32	Calculation
North Macedonia	2.09	1.86	WHO	10.30	Equal Distribution	7.08	Calculation
Northern Mariana Islands	0.05	0.09	Calculation	20.46	Equal Distribution	28.40	Calculation
Norway	5.43	12.17	WHO	18.87	Real Distribution	13.32	Calculation
Oman	4.58	7.11	WHO	11.64	Equal Distribution	13.32	Calculation
Pakistan	235.82	340.97	WHO	9.66	Equal Distribution	5.51	Calculation
Palau	0.02	0.03	Calculation	20.46	Equal Distribution	28.40	Calculation
Palestine	5.25	3.75	WHO	9.66	Equal Distribution	2.86	IDCC
Panama	4.41	8.89	WHO	10.62	Equal Distribution	19.05	Calculation
Papua New Guinea	10.14	0.74	WHO	4.00	Equal Distribution	3.90	Calculation
Paraguay	6.78	9.75	WHO	10.30	Equal Distribution	10.92	Calculation
Peru	34.05	91.41	WHO	12.94	Real Distribution	16.61	Augustovski, Federico et al. [43]
Philippines	115.56	189.32	WHO	9.40	Equal Distribution	0.95	Jiang, Yawen et al. [49]
Pitcairn	0.00	0.00	Calculation	4.00	Equal Distribution	13.32	Calculation
Poland	39.86	58.03	WHO	9.66	Real Distribution	13.32	Calculation
Portugal	10.27	28.31	WHO	17.09	Real Distribution	13.32	Calculation
Puerto Rico	3.25	5.59	Calculation	20.46	Equal Distribution	28.40	Calculation
Qatar	2.70	7.61	WHO	11.64	Equal Distribution	13.32	Calculation
Reunion	0.97	1.67	Calculation	17.95	Equal Distribution	13.32	Calculation
Romania	19.66	33.79	Calculation	16.10	Real Distribution	13.32	Calculation
Russia	144.71	187.37	WHO	10.00	Equal Distribution	13.32	Calculation
Rwanda	13.78	27.32	WHO	6.80	Real Distribution	2.16	Liu, Yang et al. [34]
Saint Barthelemy	0.01	0.02	Calculation	17.95	Equal Distribution	13.32	Calculation
Saint Helena	0.01	0.01	Calculation	4.00	Equal Distribution	13.32	Calculation
Saint Kitts and Nevis	0.05	0.06	WHO	10.62	Equal Distribution	19.05	Calculation
Saint Lucia	0.18	0.12	WHO	8.46	Equal Distribution	10.92	Calculation
Saint Martin (French part)	0.03	0.05	Calculation	17.95	Equal Distribution	13.32	Calculation
Saint Pierre and Miquelon	0.01	0.01	Calculation	17.95	Equal Distribution	13.32	Calculation
Saint Vincent and the Grenadines	0.10	0.07	WHO	9.82	Equal Distribution	10.92	Calculation
Samoa	0.22	0.45	WHO	4.00	Equal Distribution	3.90	Calculation
San Marino	0.03	0.06	Calculation	18.23	Equal Distribution	13.32	Calculation
Sao Tome and Principe	0.23	0.36	Africa CDC	9.19	Real Distribution	2.61	Calculation
Saudi Arabia	36.41	68.53	WHO	10.62	Equal Distribution	13.32	Calculation
Senegal	17.32	7.53	Africa CDC	8.90	Real Distribution	1.05	Liu, Yang et al. [34]
Serbia	6.87	8.53	WHO	9.08	Equal Distribution	7.08	Calculation
Seychelles	0.11	0.27	Africa CDC	13.69	Real Distribution	13.32	Calculation
Sierra Leone	8.61	9.16	WHO	9.30	Real Distribution	0.37	IDCC
Singapore	5.64	9.69	Calculation	17.30	Equal Distribution	2.03	Jiang, Yawen et al. [49]
Sint Maarten (Dutch part)	0.04	0.08	Calculation	15.91	Equal Distribution	13.32	Calculation
Slovakia	5.64	6.87	WHO	16.25	Real Distribution	13.32	Calculation
Slovenia	2.12	3.00	WHO	16.13	Real Distribution	13.32	Calculation
Solomon Islands	0.72	0.63	WHO	4.00	Equal Distribution	3.90	Calculation
Somalia	17.60	12.33	Africa CDC	9.49	Real Distribution	1.24	Liu, Yang et al. [34]
South Africa	59.89	41.80	WHO	11.13	Real Distribution	3.04	Edoka, I. et al. [52]
South Korea	51.82	89.06	Calculation	15.46	Real Distribution	13.32	Calculation
South Sudan	10.91	6.28	Africa CDC	9.86	Real Distribution	2.30	Calculation
Spain	47.56	112.92	WHO	17.94	Real Distribution	13.32	Lόpez, F. et al. [53]
Sri Lanka	21.83	40.12	WHO	8.15	Equal Distribution	0.39	IDCC
Sudan	46.87	26.71	Africa CDC	8.31	Real Distribution	2.82	Liu, Yang et al. [34]
Suriname	0.62	0.51	WHO	8.77	Equal Distribution	10.92	Calculation
Sweden	10.55	28.24	WHO	18.16	Real Distribution	13.32	Calculation
Switzerland	8.74	16.94	WHO	23.02	Real Distribution	13.32	Calculation
Syria	22.13	5.09	WHO	9.66	Equal Distribution	2.03	Calculation
Tajikistan	9.95	20.57	WHO	8.95	Equal Distribution	3.05	Calculation
Tanzania	65.50	43.87	Africa CDC	9.86	Real Distribution	1.04	IDCC
Thailand	71.70	142.64	WHO	10.36	Equal Distribution	0.60	Jiang, Yawen et al. [49]
Timor	1.34	2.03	WHO	8.25	Equal Distribution	3.05	Calculation
Togo	8.85	5.39	Africa CDC	8.73	Real Distribution	0.03	IDCC
Tokelau	0.00	0.00	Calculation	17.24	Equal Distribution	13.32	Calculation
Tonga	0.11	0.20	WHO	4.00	Equal Distribution	7.08	Calculation
Trinidad and Tobago	1.53	1.58	WHO	10.02	Equal Distribution	19.05	Calculation
Tunisia	12.36	13.25	WHO	7.86	Real Distribution	3.68	Liu, Yang et al. [34]
Turkey	85.34	152.54	WHO	12.32	Equal Distribution	7.08	Calculation
Turkmenistan	6.43	16.78	WHO	9.83	Equal Distribution	7.08	Calculation
Turks and Caicos Islands	0.05	0.07	WHO	17.24	Equal Distribution	13.32	Calculation
Tuvalu	0.01	0.02	Calculation	4.00	Equal Distribution	7.08	Calculation
Uganda	47.25	37.23	Africa CDC	9.06	Real Distribution	9.71	Liu, Yang et al. [34]
Ukraine	39.70	35.74	WHO	12.01	Real Distribution	7.08	Calculation
United Arab Emirates	9.44	24.92	WHO	11.64	Equal Distribution	13.32	Calculation
United Kingdom	67.51	151.25	WHO	15.94	Real Distribution	13.32	Calculation
United States	338.29	676.73	WHO	20.46	Real Distribution	28.40	Di Fusco, Manuela et al. [54]
United States Virgin Islands	0.10	0.17	Calculation	20.46	Equal Distribution	28.40	Calculation
Uruguay	3.42	9.04	WHO	7.57	Real Distribution	19.05	Calculation
Uzbekistan	34.63	83.94	WHO	9.31	Equal Distribution	3.05	Calculation
Vanuatu	0.33	0.37	WHO	4.00	Equal Distribution	3.90	Calculation
Vatican	0.00	0.00	Calculation	18.23	Equal Distribution	13.32	Calculation
Venezuela	28.30	37.86	WHO	7.23	Real Distribution	10.92	Calculation
Vietnam	98.19	266.49	WHO	8.15	Equal Distribution	1.15	IDCC
Wallis and Futuna	0.01	0.02	WHO	10.00	Equal Distribution	3.05	Calculation
Yemen	33.70	1.30	WHO	9.66	Equal Distribution	2.03	Calculation
Zambia	20.02	15.96	Africa CDC	9.67	Real Distribution	2.10	Liu, Yang et al. [34]
Zimbabwe	16.32	22.43	Africa CDC	8.89	Real Distribution	2.96	Liu, Yang et al. [34]

* Data are derived from the COVID-19 Vaccine Dashboard of the WHO [1], and the COVID-19 Vaccination Dashboard of the Africa Centre for Disease Control and Prevention (Africa CDC) [14]. Calculation means population of corresponding country multiplied by 1.7188. ** Real distribution refers to the weighted calculation based on the actual numbers of doses/proportion of vaccines from different manufacturers administered in the corresponding country. Equal distribution implies that when the real distribution cannot be obtained, it is assumed that the proportion of all manufacturers’ vaccines administered within the country is the same for the weighted calculation. Data from China are sourced from the Chinese government, and data from Cuba are assumed to be 3.

**Table A3:** Global vaccination costs.

Country/Region	Income Level	Region *	Population (Million)	Doses (Million)		Doses per Capita	Costs/Capita (USD)			Costs/Country (USD)		
				Base Value	10% Wastage		Procurement Cost	Administration Cost	Total Cost	Procurement Cost	Administration Cost	Total Cost
Afghanistan	Low income	EMR	41.13	22.96	25.26	0.56	5.56	1.13	6.69	228,487,780	46,503,619	274,991,399
Albania	Upper middle income	EUR	2.84	3.09	3.40	1.09	15.28	7.70	22.98	43,436,036	21,879,481	65,315,518
Algeria	Upper middle income	AFR	44.90	34.05	37.45	0.76	10.76	8.71	19.47	483,157,946	391,059,656	874,217,602
American Samoa	High income		0.04	0.08	0.08	1.72	38.69	48.81	87.50	1,713,619	2,162,213	3,875,831
Andorra	High income	EUR	0.08	0.16	0.17	1.97	34.42	26.20	60.62	2,748,341	2,091,905	4,840,246
Angola	Lower middle income	AFR	35.59	38.43	42.27	1.08	13.29	3.52	16.81	473,075,675	125,281,709	598,357,384
Anguilla			0.02	0.02	0.03	1.55	18.10	20.64	38.74	287,424	327,679	615,103
Antigua and Barbuda	High income	AMR	0.09	0.14	0.15	1.46	16.04	27.73	43.77	1,504,035	2,600,511	4,104,546
Argentina	Upper middle income	AMR	45.51	116.72	128.39	2.56	31.91	23.03	54.94	1,452,404,769	1,048,126,320	2,500,531,089
Armenia	Upper middle income	EUR	2.78	2.26	2.48	0.81	9.18	5.75	14.93	25,524,343	15,986,002	41,510,346
Aruba	High income		0.11	0.17	0.19	1.64	31.16	21.88	53.04	3,316,766	2,329,314	5,646,079
Australia	High income	WPR	26.18	69.69	76.66	2.66	40.42	35.45	75.88	1,058,136,715	928,096,905	1,986,233,620
Austria	High income	EUR	8.94	20.47	22.52	2.29	42.32	30.49	72.81	378,311,703	272,605,127	650,916,830
Azerbaijan	Upper middle income	EUR	10.36	13.86	15.24	1.34	15.38	9.48	24.85	159,287,491	98,151,421	257,438,912
Bahamas	High income	AMR	0.41	0.37	0.40	0.89	9.86	17.02	26.88	4,040,696	6,979,025	11,019,720
Bahrain	High income	EMR	1.47	3.48	3.82	2.36	30.25	31.45	61.70	44,531,806	46,302,234	90,834,040
Bangladesh	Lower middle income	SEAR	171.19	361.67	397.84	2.11	27.99	0.71	28.70	4,790,778,654	121,921,018	4,912,699,672
Barbados	High income	AMR	0.28	0.38	0.42	1.36	15.03	25.98	41.01	4,232,988	7,316,014	11,549,002
Belarus	Upper middle income	EUR	9.53	22.51	24.76	2.36	25.97	16.72	42.69	247,621,000	159,447,856	407,068,856
Belgium	High income	EUR	11.66	29.62	32.58	2.54	48.11	33.84	81.95	560,794,489	394,444,506	955,238,995
Belize	Upper middle income	AMR	0.41	0.51	0.56	1.26	11.72	13.76	25.48	4,749,923	5,576,178	10,326,102
Benin	Lower middle income	AFR	13.35	6.41	7.05	0.48	4.58	0.38	4.96	61,197,408	5,081,934	66,279,342
Bermuda	High income		0.06	0.13	0.15	2.06	24.02	27.39	51.41	1,542,515	1,758,552	3,301,067
Bhutan	Lower middle income	SEAR	0.78	2.01	2.21	2.57	21.74	10.03	31.77	17,009,121	7,846,753	24,855,874
Bolivia	Lower middle income	AMR	12.22	14.69	16.16	1.20	10.11	4.71	14.83	123,620,810	57,627,629	181,248,439
Bonaire Sint Eustatius and Saba			0.03	0.04	0.04	1.33	27.63	17.65	45.27	747,391	477,388	1,224,779
Bosnia and Herzegovina	Upper middle income	EUR	3.23	1.92	2.12	0.60	6.84	4.22	11.06	22,127,300	13,634,630	35,761,930
Botswana	Upper middle income	AFR	2.63	4.01	4.42	1.53	16.45	7.00	23.45	43,272,078	18,403,887	61,675,965
Brazil	Upper middle income	AMR	215.31	486.44	535.08	2.26	25.56	19.29	44.85	5,503,680,819	4,154,167,163	9,657,847,982
British Virgin Islands	High income		0.03	0.04	0.05	1.32	23.20	17.62	40.82	726,808	552,076	1,278,884
Brunei	High income	WPR	0.45	1.29	1.42	2.88	42.17	38.36	80.53	18,935,122	17,221,668	36,156,790
Bulgaria	High income	EUR	6.78	4.72	5.20	0.70	12.20	9.28	21.48	82,754,855	62,917,475	145,672,330
Burkina Faso	Low income	AFR	22.67	10.23	11.25	0.45	3.99	0.33	4.32	90,567,164	7,449,138	98,016,301
Burundi	Low income	AFR	12.89	0.80	0.88	0.06	1.00	0.14	1.15	12,944,800	1,848,748	14,793,548
Cambodia	Lower middle income	WPR	16.77	47.83	52.61	2.85	42.36	33.29	75.66	710,326,104	558,275,572	1,268,601,677
Cameroon	Lower middle income	AFR	27.91	8.24	9.06	0.30	2.87	0.46	3.32	79,985,659	12,809,577	92,795,235
Canada	High income	AMR	38.45	96.90	106.59	2.52	54.44	85.68	140.12	2,093,583,025	3,294,717,640	5,388,300,665
Cape Verde		AFR	0.59	1.09	1.20	1.83	11.87	2.12	13.98	7,039,582	1,255,180	8,294,762
Cayman Islands	High income		0.07	0.15	0.17	2.21	41.99	29.49	71.47	2,885,335	2,026,326	4,911,661
Central African Republic	Low income	AFR	5.58	3.54	3.89	0.63	5.42	1.46	6.88	30,250,979	8,145,829	38,396,808
Chad	Low income	AFR	17.72	12.15	13.36	0.69	5.78	1.58	7.36	102,492,330	27,991,259	130,483,589
Chile	High income	AMR	19.60	62.69	68.96	3.20	14.38	36.65	51.03	281,902,671	718,414,187	1,000,316,858
China	Upper middle income	WPR	1425.89	3492.33	3841.56	2.45	14.41	10.31	24.72	20,552,356,165	14,702,705,090	35,255,061,255
Colombia	Upper middle income	AMR	51.87	90.93	100.02	1.75	19.01	24.66	43.68	986,239,708	1,279,401,871	2,265,641,580
Comoros	Lower middle income	AFR	0.84	2.42	2.66	2.89	27.86	7.54	35.40	23,312,886	6,311,011	29,623,897
Congo	Lower middle income	AFR	5.97	3.46	3.80	0.58	6.33	1.69	8.02	37,774,204	10,092,414	47,866,617
Cook Islands		WPR	0.02	0.04	0.04	2.40	45.49	31.95	77.43	774,755	544,099	1,318,854
Costa Rica	Upper middle income	AMR	5.18	13.58	14.93	2.62	25.29	28.70	53.99	131,026,474	148,667,438	279,693,912
Cote d’Ivoire	Lower middle income	AFR	28.16	33.64	37.00	1.19	12.88	0.53	13.41	362,676,399	15,024,629	377,701,029
Croatia	High income	EUR	4.03	5.42	5.96	1.34	24.29	17.90	42.19	97,893,002	72,142,794	170,035,796
Cuba	Upper middle income	AMR	11.21	45.62	50.18	4.07	13.43	44.43	57.85	150,552,679	498,116,465	648,669,145
Curacao	High income		0.19	0.26	0.29	1.36	23.83	18.14	41.96	4,554,976	3,467,028	8,022,003
Cyprus	High income	EUR	0.90	1.80	1.98	2.01	35.86	26.73	62.60	32,134,175	23,954,114	56,088,288
Czechia	High income	EUR	10.49	19.03	20.93	1.81	34.37	24.15	58.51	360,637,590	253,384,502	614,022,092
Democratic Republic of Congo	Low income	AFR	99.01	36.83	40.51	0.37	2.82	0.94	3.76	278,885,937	93,173,600	372,059,537
Denmark	High income	EUR	5.88	14.96	16.46	2.54	50.99	33.87	84.86	299,934,592	199,259,360	499,193,952
Djibouti	Lower middle income	EMR	1.12	2.22	2.44	1.98	30.25	10.90	41.15	33,901,577	12,220,403	46,121,979
Dominica	Upper middle income	AMR	0.07	0.07	0.07	0.93	9.29	10.15	19.44	675,658	738,592	1,414,251
Dominican Republic	Upper middle income	AMR	11.23	16.43	18.07	1.46	16.82	15.98	32.80	188,866,747	179,391,918	368,258,665
Ecuador	Upper middle income	AMR	18.00	39.56	43.52	2.20	30.57	24.00	54.57	550,333,001	431,975,069	982,308,070
Egypt	Lower middle income	EMR	110.99	112.67	123.94	1.02	9.36	8.92	18.28	1,038,850,916	989,837,005	2,028,687,921
El Salvador	Upper middle income	AMR	6.34	11.29	12.42	1.78	20.48	19.45	39.93	129,769,067	123,258,976	253,028,042
Equatorial Guinea	Upper middle income	AFR	1.67	1.22	1.34	0.73	12.20	4.29	16.50	20,438,000	7,191,900	27,629,900
Eritrea	Low income	AFR	3.68	6.33	6.97	1.72	12.53	3.96	16.49	46,148,874	14,589,376	60,738,250
Estonia	High income	EUR	1.33	2.20	2.42	1.66	30.17	22.11	52.28	40,012,462	29,315,551	69,328,013
Eswatini	Lower middle income	AFR	1.20	0.91	1.00	0.76	4.94	3.01	7.96	5,941,392	3,622,345	9,563,736
Ethiopia	Low income	AFR	123.38	81.10	89.20	0.66	7.10	1.46	8.55	875,861,036	179,625,425	1,055,486,461
Faeroe Islands	High income		0.05	0.10	0.11	1.96	39.21	26.05	65.26	2,082,770	1,383,673	3,466,444
Falkland Islands			0.00	0.00	0.00	1.16	20.33	15.44	35.77	77,269	58,693	135,962
Fiji	Upper middle income	WPR	0.93	1.56	1.71	1.67	16.14	11.85	27.99	15,008,543	11,015,523	26,024,066
Finland	High income	EUR	5.54	13.33	14.66	2.41	48.02	32.03	80.05	266,080,780	177,472,878	443,553,658
France	High income	EUR	67.81	154.49	169.94	2.28	44.98	30.34	75.32	3,050,071,773	2,057,560,696	5,107,632,469
French Guiana			0.30	0.52	0.58	1.72	33.93	22.89	56.82	10,334,991	6,971,925	17,306,917
French Polynesia	High income		0.31	0.50	0.55	1.64	32.42	21.87	54.29	9,929,492	6,698,378	16,627,870
Gabon	Upper middle income	AFR	2.39	2.20	2.42	0.92	8.00	5.44	13.44	19,114,447	12,990,399	32,104,845
Gambia	Low income	AFR	2.71	1.81	1.99	0.67	5.24	1.54	6.78	14,166,537	4,178,416	18,344,953
Georgia	Upper middle income	EUR	3.74	2.93	3.22	0.78	9.00	5.54	14.54	33,688,132	20,758,303	54,446,435
Germany	High income	EUR	83.37	192.22	211.44	2.31	44.94	30.71	75.65	3,746,604,407	2,560,029,623	6,306,634,030
Ghana	Lower middle income	AFR	33.48	32.35	35.59	0.97	6.28	3.02	9.30	210,122,850	101,107,906	311,230,756
Gibraltar	High income		0.03	0.13	0.15	4.06	47.48	54.13	101.61	1,551,486	1,768,780	3,320,267
Greece	High income	EUR	10.38	22.30	24.53	2.15	33.40	28.60	62.00	346,838,366	296,980,639	643,819,006
Greenland	High income		0.06	0.08	0.09	1.41	41.11	18.80	59.91	2,322,608	1,061,961	3,384,569
Grenada	Upper middle income	AMR	0.13	0.09	0.10	0.72	6.72	7.89	14.61	843,425	990,140	1,833,565
Guadeloupe			0.40	0.68	0.75	1.72	33.93	22.89	56.82	13,429,503	9,059,465	22,488,968
Guam	High income		0.17	0.30	0.32	1.72	38.69	48.81	87.50	6,645,684	8,385,402	15,031,086
Guatemala	Upper middle income	AMR	17.84	20.36	22.40	1.14	11.51	12.46	23.97	205,329,828	222,320,239	427,650,067
Guernsey			0.06	0.18	0.20	2.82	49.43	37.54	86.97	3,130,131	2,377,618	5,507,749
Guinea	Lower middle income	AFR	13.86	12.05	13.25	0.87	9.62	2.27	11.89	133,346,800	31,407,945	164,754,745
Guinea-Bissau	Low income	AFR	2.11	1.76	1.94	0.84	5.69	1.93	7.62	11,975,040	4,064,297	16,039,337
Guyana	High income	AMR	0.81	0.96	1.06	1.19	17.86	2.78	20.63	14,441,040	2,245,514	16,686,554
Haiti	Lower middle income	AMR	11.59	0.68	0.75	0.06	0.56	0.05	0.61	6,490,448	623,770	7,114,218
Honduras	Lower middle income	AMR	10.43	17.07	18.78	1.64	12.59	11.34	23.93	131,381,569	118,301,123	249,682,692
Hong Kong, China	High income		7.49	20.92	23.02	2.79	30.38	4.69	35.07	227,492,447	35,153,466	262,645,913
Hungary	High income	EUR	9.97	16.70	18.37	1.68	29.71	22.32	52.03	296,162,106	222,429,183	518,591,288
Iceland	High income	EUR	0.37	0.81	0.89	2.16	42.66	28.77	71.42	15,906,552	10,727,337	26,633,889
India	Lower middle income	SEAR	1417.17	2206.75	2427.43	1.56	6.66	4.46	11.12	9,444,165,919	6,313,862,476	15,758,028,394
Indonesia	Upper middle income	SEAR	275.50	448.19	493.01	1.63	17.69	0.98	18.66	4,872,599,158	268,915,483	5,141,514,641
Iran	Upper middle income	EMR	88.55	155.46	171.01	1.76	20.67	14.34	35.01	1,830,517,771	1,269,809,692	3,100,327,463
Iraq	Upper middle income	EMR	44.50	19.56	21.51	0.44	4.67	2.61	7.28	207,912,164	115,994,238	323,906,402
Ireland	High income	EUR	5.02	13.05	14.35	2.60	48.89	34.59	83.49	245,600,841	173,769,014	419,369,855
Isle of Man	High income		0.08	0.19	0.21	2.25	39.41	29.93	69.34	3,331,220	2,530,364	5,861,584
Israel	High income	EUR	9.45	18.65	20.51	1.97	37.43	26.29	63.71	353,664,339	248,373,024	602,037,363
Italy	High income	EUR	59.04	144.61	159.07	2.45	49.12	32.62	81.74	2,900,061,204	1,925,918,658	4,825,979,861
Jamaica	Upper middle income	AMR	2.83	1.53	1.68	0.54	5.03	5.90	10.93	14,215,303	16,688,072	30,903,375
Japan	High income	WPR	123.95	383.75	422.12	3.10	65.52	41.23	106.75	8,120,698,268	5,110,800,512	13,231,498,780
Jersey			0.11	0.27	0.29	2.41	42.24	32.09	74.33	4,680,565	3,555,314	8,235,878
Jordan	Lower middle income	EMR	11.29	10.06	11.06	0.89	9.47	4.91	14.38	106,925,215	55,402,679	162,327,894
Kazakhstan	Upper middle income	EUR	19.40	38.36	42.19	1.98	25.99	14.01	40.00	504,184,428	271,676,913	775,861,341
Kenya	Lower middle income	AFR	54.03	32.27	35.49	0.60	3.86	4.81	8.67	208,641,829	259,911,893	468,553,722
Kiribati	Lower middle income	WPR	0.13	0.22	0.24	1.68	7.38	6.53	13.91	967,912	857,352	1,825,264
Kosovo	Upper middle income		1.78	1.84	2.02	1.03	9.25	7.30	16.56	16,492,915	13,014,829	29,507,745
Kuwait	High income	EMR	4.27	8.26	9.09	1.94	24.79	25.77	50.56	105,817,062	110,023,978	215,841,039
Kyrgyzstan	Lower middle income	EUR	6.63	3.66	4.03	0.55	6.97	1.69	8.66	46,215,201	11,184,233	57,399,434
Laos	Lower middle income	WPR	7.53	11.11	12.22	1.48	15.26	2.67	17.93	114,888,422	20,138,947	135,027,369
Latvia	High income	EUR	1.85	2.90	3.19	1.56	30.08	20.84	50.92	55,658,716	38,571,329	94,230,045
Lebanon	Lower middle income	EMR	5.49	5.81	6.40	1.06	11.26	5.83	17.09	61,815,419	32,029,300	93,844,719
Lesotho	Lower middle income	AFR	2.31	3.43	3.77	1.49	12.36	3.87	16.23	28,500,299	8,929,420	37,429,718
Liberia	Low income	AFR	5.30	5.35	5.89	1.01	7.93	1.63	9.56	42,049,887	8,641,968	50,691,855
Libya	Upper middle income	EMR	6.81	7.11	7.82	1.04	13.42	10.85	24.28	91,434,449	73,947,503	165,381,952
Liechtenstein	High income		0.04	0.07	0.08	1.89	48.83	25.16	73.99	1,921,736	990,029	2,911,765
Lithuania	High income	EUR	2.75	4.60	5.06	1.67	29.08	22.29	51.37	79,974,448	61,304,557	141,279,004
Luxembourg	High income	EUR	0.65	1.37	1.51	2.12	42.93	28.27	71.20	27,803,631	18,306,389	46,110,021
Madagascar	Low income	AFR	29.61	15.66	17.22	0.53	4.12	1.25	5.36	121,907,913	36,874,378	158,782,291
Malawi	Low income	AFR	20.41	10.93	12.02	0.54	3.88	0.78	4.66	79,140,573	16,013,402	95,153,975
Malaysia	Upper middle income	WPR	33.94	72.65	79.91	2.14	26.75	15.16	41.91	907,797,287	514,566,798	1,422,364,085
Maldives	Upper middle income	SEAR	0.52	0.95	1.05	1.82	19.79	1.09	20.88	10,364,710	571,058	10,935,768
Mali	Low income	AFR	22.59	8.44	9.28	0.37	2.29	0.51	2.79	51,689,930	11,441,506	63,131,436
Malta	High income	EUR	0.53	1.41	1.55	2.64	48.46	35.16	83.62	25,842,156	18,749,923	44,592,079
Marshall Islands	Upper middle income	WPR	0.04	0.07	0.08	1.72	38.69	48.81	87.50	1,609,088	2,030,317	3,639,405
Martinique			0.37	0.63	0.69	1.72	33.93	22.89	56.82	12,470,888	8,412,788	20,883,676
Mauritania	Lower middle income	AFR	4.74	7.64	8.40	1.61	15.66	4.20	19.87	74,182,009	19,910,135	94,092,144
Mauritius	Upper middle income	AFR	1.30	3.39	3.72	2.61	37.10	15.36	52.46	48,214,415	19,962,062	68,176,477
Mayotte			0.33	0.56	0.62	1.72	33.93	22.89	56.82	11,066,084	7,465,116	18,531,200
Mexico	Upper middle income	AMR	127.50	223.16	245.47	1.75	20.43	11.13	31.57	2,605,540,643	1,419,291,195	4,024,831,838
Micronesia (country)	Lower middle income	WPR	0.11	0.20	0.22	1.72	38.69	48.81	87.50	4,417,148	5,573,476	9,990,624
Moldova	Upper middle income	EUR	3.27	2.29	2.52	0.70	7.63	1.75	9.38	24,971,707	5,718,409	30,690,116
Monaco	High income	EUR	0.04	0.06	0.07	1.72	32.60	22.89	55.49	1,189,436	835,323	2,024,758
Mongolia	Upper middle income	WPR	3.40	5.49	6.04	1.62	16.14	11.45	27.59	54,853,267	38,899,844	93,753,111
Montenegro	Upper middle income	EUR	0.63	0.68	0.75	1.08	10.82	7.67	18.49	6,783,560	4,810,642	11,594,202
Montserrat			0.00	0.00	0.01	1.05	4.61	13.94	18.55	20,324	61,516	81,840
Morocco	Lower middle income	EMR	37.46	60.80	66.88	1.62	28.19	6.60	34.79	1,056,095,057	247,138,098	1,303,233,154
Mozambique	Low income	AFR	32.97	48.95	53.84	1.48	13.43	2.04	15.47	442,714,197	67,302,700	510,016,897
Myanmar	Lower middle income	SEAR	54.18	93.48	102.82	1.73	13.29	2.98	16.26	719,773,701	161,358,269	881,131,970
Namibia	Upper middle income	AFR	2.57	2.09	2.29	0.81	8.88	3.64	12.51	22,785,101	9,336,193	32,121,293
Nauru	High income	WPR	0.01	0.03	0.04	2.53	11.15	33.75	44.89	141,491	428,271	569,762
Nepal	Lower middle income	SEAR	30.55	62.63	68.89	2.05	26.41	2.34	28.75	806,659,327	71,452,061	878,111,388
Netherlands	High income	EUR	17.56	39.76	43.74	2.26	47.20	30.15	77.36	829,108,197	529,584,364	1,358,692,561
New Caledonia	High income		0.29	0.48	0.53	1.66	31.44	22.08	53.52	9,116,033	6,402,050	15,518,082
New Zealand	High income	WPR	5.19	12.35	13.58	2.38	27.82	31.71	59.53	144,236,462	164,437,536	308,673,999
Nicaragua	Lower middle income	AMR	6.95	15.51	17.06	2.23	21.46	8.76	30.22	149,140,372	60,833,450	209,973,822
Niger	Low income	AFR	26.21	11.30	12.43	0.43	3.95	1.75	5.70	103,463,602	45,819,413	149,283,015
Nigeria	Lower middle income	AFR	218.54	133.05	146.35	0.61	4.56	1.60	6.16	996,232,841	350,581,535	1,346,814,377
Niue		WPR	0.00	0.00	0.01	2.52	47.83	33.59	81.42	93,360	65,565	158,925
North Macedonia	Upper middle income	EUR	2.09	1.86	2.05	0.89	10.08	6.30	16.38	21,107,643	13,195,739	34,303,382
Northern Mariana Islands	High income		0.05	0.09	0.09	1.72	38.69	48.81	87.50	1,917,845	2,419,901	4,337,746
Norway	High income	EUR	5.43	12.17	13.38	2.24	46.48	29.82	76.30	252,607,338	162,045,068	414,652,407
Oman	High income	EMR	4.58	7.11	7.82	1.55	19.90	20.69	40.58	91,052,589	94,672,521	185,725,109
Pakistan	Lower middle income	EMR	235.82	340.97	375.07	1.45	15.37	7.96	23.34	3,624,858,239	1,878,199,243	5,503,057,482
Palau	High income	WPR	0.02	0.03	0.03	1.72	38.69	48.81	87.50	699,607	882,751	1,582,358
Palestine			5.25	3.75	4.12	0.71	7.59	2.04	9.63	39,850,642	10,723,332	50,573,973
Panama	High income	AMR	4.41	8.89	9.78	2.02	23.56	38.43	61.99	103,888,038	169,408,896	273,296,934
Papua New Guinea	Lower middle income	WPR	10.14	0.74	0.81	0.07	0.32	0.28	0.60	3,245,858	2,875,100	6,120,958
Paraguay	Upper middle income	AMR	6.78	9.75	10.72	1.44	16.28	15.69	31.98	110,417,025	106,405,109	216,822,134
Peru	Upper middle income	AMR	34.05	91.41	100.55	2.68	38.23	44.59	82.82	1,301,634,222	1,518,362,954	2,819,997,175
Philippines	Lower middle income	WPR	115.56	189.32	208.25	1.64	16.94	1.56	18.50	1,957,539,414	179,851,300	2,137,390,714
Pitcairn			0.00	0.00	0.00	1.72	7.56	22.89	30.45	355	1,076	1,431
Poland	High income	EUR	39.86	58.03	63.84	1.46	15.47	19.39	34.86	616,649,541	772,879,347	1,389,528,888
Portugal	High income	EUR	10.27	28.31	31.15	2.76	51.84	36.72	88.55	532,421,960	377,100,018	909,521,978
Puerto Rico	High income		3.25	5.59	6.15	1.72	38.69	48.81	87.50	125,824,461	158,762,979	284,587,440
Qatar	High income	EMR	2.70	7.61	8.37	2.82	36.16	37.60	73.76	97,465,116	101,339,987	198,805,103
Reunion			0.97	1.67	1.84	1.72	33.93	22.89	56.82	33,053,119	22,297,442	55,350,561
Romania	High income	EUR	19.66	33.79	37.17	1.72	30.44	22.89	53.33	598,422,127	450,024,164	1,048,446,291
Russia	High income	EUR	144.71	187.37	206.11	1.29	14.24	17.24	31.49	2,061,119,588	2,495,477,202	4,556,596,790
Rwanda	Low income	AFR	13.78	27.32	30.05	1.98	14.84	4.27	19.12	204,491,437	58,879,037	263,370,474
Saint Barthelemy			0.01	0.02	0.02	1.72	33.93	22.89	56.82	373,062	251,666	624,728
Saint Helena			0.01	0.01	0.01	1.72	7.56	22.89	30.45	40,846	123,635	164,482
Saint Kitts and Nevis	High income	AMR	0.05	0.06	0.07	1.27	14.81	24.16	38.97	706,375	1,151,877	1,858,253
Saint Lucia	Upper middle income	AMR	0.18	0.12	0.14	0.68	6.36	7.46	13.82	1,143,748	1,342,704	2,486,451
Saint Martin (French part)	High income		0.03	0.05	0.06	1.72	33.93	22.89	56.82	1,079,621	728,306	1,807,927
Saint Pierre and Miquelon			0.01	0.01	0.01	1.72	33.93	22.89	56.82	199,697	134,715	334,412
Saint Vincent and the Grenadines	Upper middle income	AMR	0.10	0.07	0.08	0.71	7.63	7.71	15.34	793,129	801,875	1,595,004
Samoa	Lower middle income	WPR	0.22	0.45	0.50	2.04	8.96	7.94	16.90	1,993,565	1,765,850	3,759,416
San Marino	High income	EUR	0.03	0.06	0.06	1.72	34.47	22.89	57.36	1,161,285	771,204	1,932,489
Sao Tome and Principe	Lower middle income	AFR	0.23	0.36	0.40	1.58	16.00	4.12	20.13	3,639,116	937,976	4,577,092
Saudi Arabia	High income	EMR	36.41	68.53	75.39	1.88	21.99	25.07	47.06	800,621,559	912,752,813	1,713,374,372
Senegal	Lower middle income	AFR	17.32	7.53	8.29	0.43	4.26	0.45	4.72	73,781,793	7,871,335	81,653,128
Serbia	Upper middle income	EUR	6.87	8.53	9.39	1.24	12.41	8.80	21.20	85,244,464	60,452,121	145,696,585
Seychelles	High income	AFR	0.11	0.27	0.30	2.53	38.11	33.70	71.81	4,082,716	3,610,810	7,693,526
Sierra Leone	Low income	AFR	8.61	9.16	10.07	1.06	10.89	0.39	11.28	93,712,936	3,375,379	97,088,315
Singapore	High income	WPR	5.64	9.69	10.66	1.72	32.71	3.49	36.20	184,380,022	19,668,494	204,048,516
Sint Maarten (Dutch part)	High income		0.04	0.08	0.08	1.72	30.07	22.89	52.97	1,329,049	1,011,608	2,340,656
Slovakia	High income	EUR	5.64	6.87	7.56	1.22	21.77	16.21	37.98	122,831,694	91,498,172	214,329,866
Slovenia	High income	EUR	2.12	3.00	3.30	1.41	25.08	18.83	43.90	53,155,773	39,909,667	93,065,441
Solomon Islands	Lower middle income	WPR	0.72	0.63	0.69	0.86	3.80	3.37	7.17	2,754,206	2,439,608	5,193,814
Somalia	Low income	EMR	17.60	12.33	13.56	0.70	7.31	0.87	8.17	128,613,012	15,222,437	143,835,449
South Africa	Upper middle income	AFR	59.89	41.80	45.98	0.70	8.55	2.12	10.66	511,905,690	126,859,394	638,765,085
South Korea	High income	WPR	51.82	89.06	97.97	1.72	29.22	22.89	52.11	1,514,227,220	1,186,125,714	2,700,352,933
South Sudan	Low income	AFR	10.91	6.28	6.91	0.58	6.24	1.33	7.57	68,102,474	14,470,857	82,573,331
Spain	High income	EUR	47.56	112.92	124.22	2.37	46.85	31.62	78.48	2,228,302,490	1,503,942,803	3,732,245,293
Sri Lanka	Lower middle income	SEAR	21.83	40.12	44.13	1.84	16.47	0.72	17.19	359,645,229	15,671,425	375,316,654
Sudan	Low income	EMR	46.87	26.71	29.38	0.57	5.21	1.60	6.81	244,110,900	75,196,701	319,307,601
Suriname	Upper middle income	AMR	0.62	0.51	0.56	0.82	7.90	8.93	16.83	4,880,332	5,521,390	10,401,723
Sweden	High income	EUR	10.55	28.24	31.06	2.68	53.48	35.65	89.13	564,151,428	376,074,882	940,226,310
Switzerland	High income	EUR	8.74	16.94	18.63	1.94	49.07	25.81	74.88	428,906,725	225,618,581	654,525,306
Syria	Low income	EMR	22.13	5.09	5.60	0.23	2.45	0.47	2.91	54,117,922	10,308,526	64,426,448
Tajikistan	Lower middle income	EUR	9.95	20.57	22.62	2.07	20.35	6.31	26.66	202,493,565	62,829,303	265,322,868
Tanzania	Lower middle income	AFR	65.50	43.87	48.26	0.67	7.27	0.69	7.96	476,012,130	45,425,136	521,437,266
Thailand	Upper middle income	SEAR	71.70	142.64	156.90	1.99	22.67	1.19	23.87	1,625,468,620	85,581,008	1,711,049,628
Timor	Lower middle income		1.34	2.03	2.23	1.51	13.73	4.62	18.35	18,411,342	6,197,343	24,608,685
Togo	Low income	AFR	8.85	5.39	5.93	0.61	5.85	0.02	5.86	51,744,682	139,926	51,884,608
Tokelau			0.00	0.00	0.00	1.72	32.60	22.89	55.49	61,703	43,333	105,036
Tonga	Upper middle income	WPR	0.11	0.20	0.22	1.90	8.37	13.47	21.84	894,115	1,439,344	2,333,460
Trinidad and Tobago	High income	AMR	1.53	1.58	1.74	1.03	11.38	19.67	31.05	17,424,658	30,115,614	47,540,272
Tunisia	Lower middle income	EMR	12.36	13.25	14.58	1.07	9.24	3.94	13.18	114,192,967	48,705,940	162,898,907
Turkey	Upper middle income	EUR	85.34	152.54	167.80	1.79	24.22	12.66	36.88	2,067,267,357	1,080,481,040	3,147,748,397
Turkmenistan	Upper middle income	EUR	6.43	16.78	18.45	2.61	28.20	18.48	46.68	181,337,052	118,826,016	300,163,068
Turks and Caicos Islands	High income		0.05	0.07	0.08	1.62	30.65	21.53	52.18	1,401,572	984,303	2,385,875
Tuvalu	Upper middle income	WPR	0.01	0.02	0.02	1.72	7.56	12.17	19.74	85,723	137,997	223,721
Uganda	Low income	AFR	47.25	37.23	40.96	0.79	7.86	7.65	15.51	371,198,696	361,529,323	732,728,019
Ukraine	Upper middle income	EUR	39.70	35.74	39.31	0.90	11.89	6.38	18.27	472,111,285	253,118,340	725,229,625
United Arab Emirates	High income	EMR	9.44	24.92	27.41	2.64	33.81	35.16	68.97	319,223,821	331,915,041	651,138,862
United Kingdom	High income	EUR	67.51	151.25	166.37	2.24	39.28	29.84	69.12	2,651,889,556	2,014,350,758	4,666,240,313
United States	High income	AMR	338.29	676.73	744.40	2.00	45.03	56.81	101.84	15,231,715,711	19,219,097,409	34,450,813,120
United States Virgin Islands			0.10	0.17	0.19	1.72	38.69	48.81	87.50	3,848,495	4,855,960	8,704,455
Uruguay	High income	AMR	3.42	9.04	9.94	2.64	21.98	50.29	72.27	75,233,771	172,138,183	247,371,954
Uzbekistan	Lower middle income	EUR	34.63	83.94	92.33	2.42	24.84	7.40	32.24	860,004,294	256,404,190	1,116,408,484
Vanuatu	Lower middle income	WPR	0.33	0.37	0.40	1.12	4.94	4.37	9.31	1,613,207	1,428,939	3,042,146
Vatican			0.00	0.00	0.00	1.72	34.47	22.89	57.36	27,852	18,496	46,348
Venezuela		AMR	28.30	37.86	41.65	1.34	10.64	14.61	25.24	301,007,329	413,378,953	714,386,282
Vietnam	Lower middle income	WPR	98.19	266.49	293.14	2.71	24.33	3.13	27.46	2,389,102,116	307,593,527	2,696,695,643
Wallis and Futuna	Lower middle income		0.01	0.02	0.02	1.56	17.13	4.76	21.89	198,638	55,162	253,800
Yemen	Low income	EMR	33.70	1.30	1.43	0.04	0.41	0.08	0.49	13,805,846	2,629,774	16,435,621
Zambia	Lower middle income	AFR	20.02	15.96	17.56	0.80	8.48	1.67	10.15	169,708,303	33,439,447	203,147,750
Zimbabwe	Lower middle income	AFR	16.32	22.43	24.68	1.37	13.44	4.06	17.51	219,407,705	66,288,924	285,696,629
AFR			1191.65	835.11	918.62	0.70	6.62 [4.43–8.81]	2.23 [0.73–3.73]	8.85 [5.34–12.37]	7,891,058,293	2,660,258,729	10,551,317,022
AMR			1032.01	2111.70	2322.87	2.05	30.89 [27.17–34.62]	34.34 [28.53–40.14]	65.23 [56.18–74.28]	31,883,410,037	35,436,093,464	67,319,503,500
EMR			774.67	916.22	1007.84	1.18	13.29 [8.67–17.90]	8.35 [3.30–13.41]	21.64 [12.31–30.96]	10,294,351,184	6,470,151,731	16,764,502,915
EUR			933.96	1775.10	1952.61	1.90	31.28 [27.41–35.14]	22.09 [19.31–24.87]	53.36 [46.79–59.94]	29,210,812,524	20,628,974,777	49,839,787,301
WPR			1927.28	4655.60	5121.16	2.42	19.59 [13.39–25.79]	12.34 [6.20–18.47]	31.93 [20.35–43.51]	37,761,775,163	23,779,866,101	61,541,641,264
SEAR			2043.42	3358.44	3694.28	1.64	11.08 [7.01–15.16]	3.45 [1.59–5.31]	14.53 [10.31–18.75]	22,646,464,439	7,047,179,552	29,693,643,991
Low income			677.66	408.95	449.84	0.60	5.55 [4.15–6.96]	1.65 [1.02–2.27]	7.2 [5.38–9.02]	3,762,644,482	1,115,415,035	4,878,059,517
Lower middle income			3013.04	4392.40	4831.64	1.46	10.8 [8.18–13.41]	4.22 [2.02–6.42]	15.02 [10.64–19.40]	32,537,014,829	12,714,535,815	45,251,550,644
Upper middle income			2820.43	5908.32	6499.15	2.09	17.22 [14.88–19.56]	10.99 [8.26–13.71]	28.21 [23.60–32.83]	48,574,069,949	30,983,712,802	79,557,782,750
High income			1378.54	2936.90	3230.59	2.13	39.86 [37.28–42.45]	37.03 [34.39–39.68]	76.9 [72.38–81.41]	54,951,016,292	51,053,460,948	106,004,477,240
World			7926.55	13,694.11	15,063.52	1.73	17.70 [15.84–19.55]	12.16 [10.29–14.02]	29.85 [26.33–33.37]	140,267,360,919	96,359,585,554	236,626,946,473

* Full names for different regions are African Region (AFR), the Region of the Americas (AMR), the Eastern Mediterranean Region (EMR), the European Region (EUR), the South-East Asia Region (SEAR), and the Western Pacific Region (WPR).
