# Supplementary material for: Estimating the Global, Regional, and National Economic Costs of COVID-19 Vaccination During the COVID-19 Pandemic
Source: Vaccines (Basel). 2025 Nov 11;13(11):1153. doi: 10.3390/vaccines13111153 (PMC12657000; doi:10.3390/vaccines13111153)
Supplement: Supplementary file 1 [file vaccines-13-01153-s001.zip › vaccines-3970403-supplementary.pdf]

Supplementary Material

S1 Estimates of the average price of COVID-19 vaccines in each country

The average procurement cost for COVID-19 vaccine per dose in each country was estimated using a weighted average of vaccine prices and the corresponding number of doses supplied by each manufacturer. Pricing data for COVID-19 vaccines from various manufacturers were obtained from MI4A and public market sources. For the MI4A report, we collected the average price for countries within the same income group. The detailed vaccine prices and data sources are provided in Table S1. The quantities of vaccine supplied by the different manufacturers in a given country were derived from a variety of data sources, including Our World in Data, the African Centers for Disease Control and Prevention (Africa CDC), the Pan American Health Organization (PAHO), and pertinent literature, as outlined in Table S2.

Estimates of average vaccine prices can be divided into several categories, depending on the availability of data for different countries. First of all, for countries with data on vaccine doses and vaccine prices by manufacturer, we used the weighted average method for estimation. In some countries, vaccination against COVID-19 involved vaccines from multiple manufacturers, but no specific data were available on the doses of vaccine supplied by each manufacturer. Procurement costs were therefore conservatively estimated using the lowest value of vaccine prices among the vaccines administered. For countries that reported using only one vaccine, the price of the corresponding vaccine was adopted directly. For island countries where no average vaccine prices were available, it was assumed that the vaccine procurement price per dose would be consistent with its parent country. For instance, vaccine procurement cost per dose from France were used as a proxy for French Polynesia. In addition, vaccine procurement costs in China were obtained from official public information from the Chinese Center for Disease Control and Prevention as well as from government agencies. In the case of Cuba, the vaccine was developed independently and no data on the price of the vaccine could be found. Considering that the Cuban vaccine is a protein subunit vaccine that can be produced on a large scale at low production costs and has relatively less stringent cold-chain storage requirements, the price of the vaccine in Cuba is assumed to be relatively low. An average vaccine procurement cost of US$ 3 per dose was used to estimate the cost of COVID-19 vaccination in Cuba[1].

Table S1 Vaccine Price Input

Vaccine	Price value	Source
CanSino	Upper middle income：15	Mi4A[2]
CNBG	Low income:4<br>Lower middle income:11.25<br>Upper middle income:8.36	Mi4A[2]
Corbevax	1.92	Public Market Information[3]
Covaxin	3.53	Public Market Information[4]
Covishield	3	Public Market Information[4]
Johnson&Johnson	Low income:7.5<br>Lower middle income:7.5<br>Upper middle income:7.5<br>High income:8.84	Mi4A[2]
Moderna	Low income:10<br>Lower middle income:10<br>Upper middle income:10<br>High income:26.48	Mi4A[2]
Novavax	9.51	Public Market Information[5]
Oxford/AstraZeneca	4	Mi4A[2]
Pfizer/BioNTech	Low income:7<br>Lower middle income:6.75<br>Upper middle income:12.32<br>High income:17.24	Mi4A[2]
Sinopharm/Beijing	19	Public Market Information[6]
Sinovac	Lower middle income:14<br>Upper middle income:15.48	Mi4A[2]
Sputnik light	10	Public Market Information[6]
Sputnik V	10	Public Market Information[4]

Table S2 Country-Level Vaccine Doses by Manufacturer

**Country**	Pfizer/BioNTech	Moderna	Oxford/AstraZeneca	Johnson&Johnson	Sputnik V	Sinovac	Sinopharm/Beijing	CanSino	Novavax	Covaxin	CNBG	Sputnik light	Corbevax	covishield	Source
Afghanistan*			0.15	0.67			0.18								Hamidullah Matin et al[7]
Albania	735635		249200		60000	1000000									Public Resource[8-14]
Algeria			2865600	6508800	1000000	22975200	700000								Africa CDC
Angola	10571310		7208440	4972050	588550		15089622								Africa CDC
Argentina	20698755	18470054	26782388	0	20758838	0	28986044	989067	0	0					Ourworldindata
Australia	125000000	25000000	53800000						51000000						Public Resource[15-18]
Austria	16813415	1666123	1593253	368544	0	0	0	0	15073	0					Ourworldindata
Bangladesh	80624776	15808126	56280768	607263	0	61308026	114148258								Ourworldindata
Belgium	21947093	4389520	2849295	428653	0	0	0	0	2558	0					Ourworldindata
Benin	1107990		278400	3665850	100000	1253400						100000			Africa CDC
Botswana	1108080		574560	1396800		804494				130000					Africa CDC
Brazil*	0.42		0.3	0.04		0.22									PAHO
Bulgaria	3094606	511980	478547	531805	0	0	0	0	0	0					Ourworldindata
Burkina Faso	2704230		246800	5600450		600000	1074400								Africa CDC
Burundi				302400			500000								Africa CDC
Cambodia	2489760	188160	3168040	1060100		29424800	7800000							324000	Ikuma Nozaki et al[19]
Cameroon	264420		762850	6010400			1200000								Africa CDC
Canada	63482165	28804585	2815498	23591	0	0	0	0	28433	0					Ourworldindata
Cape Verde	433260		604200				50000								Africa CDC
Central African Republic			160160	3074700		100620	200000								Africa CDC
Chad	483210			11465650			200000								Africa CDC
Chile	8067724	0	549673	0	0	25943395	0	0	0	0					Ourworldindata
Comoros	2003190		18000				400000								Africa CDC

Congo	809500			1772800	12000		760000				102006	Africa CDC
Cote d'Ivoire	12879090		4671340	7092400			8994400					Africa CDC
Croatia	4113747	527453	568661	205651	0	0	0	0	1376	0		Ourworldindata
Cyprus	1309713	199722	254531	31066	0	0	0	0	901	0		Ourworldindata
Czechia	16067298	1643596	887777	415092	23	370	90	0	11278	10		Ourworldindata
Democratic Republic of Congo	5828310		1817000	26382200		2400000	400000					Africa CDC
Denmark	12137298	1767250	155574	45378	0	0	0	0	0	0		Ourworldindata
Djibouti	74880		175200	468450	100000	300000	1100000					Africa CDC
Ecuador	8552679	0	5009163	0	0	15812935	0	536882	0	0		Ourworldindata
Egypt	29770090		29190320	15513450	393000	20600000	6950000					Africa CDC
Equatorial Guinea				400000			820000					Africa CDC
Estonia	1637131	244655	238121	79103	0	0	0	0	2167	0		Ourworldindata
Eswatini	250380		360800	302400								Africa CDC
Ethiopia	12061260		6981190	42062150		600000	19390400					Africa CDC
Finland	10288601	2068483	553922	0	0	0	0	0	0	0		Ourworldindata
France	121360954	24133395	7863058	1091088	0	0	0	0	39950	0		Ourworldindata
Gabon	420030		350000	1413600	20000							Africa CDC
Gambia	819810		89400	904320								Africa CDC
Germany	138203939	31586566	12803142	3761436	0	0	0	0	160154	0		Ourworldindata
Ghana	9831510		12662020	9840000	21000							Africa CDC
Guatemala*	0.21	0.42	0.22		0.15							PAHO
Guinea	3001650		1001320	3388800	407990	1506400	2100000					Africa CDC
Guinea-Bissau			669600	1094400								Africa CDC
Haiti*	0.15	0.52		0.34								PAHO
Honduras*	0.62	0.21	0.16									PAHO
Hong Kong	11996016	0	0	0	0	8928666	0	0	0	0		Ourworldindata
Hungary	9783019	1079207	1252978	346000	1807392	0	2315511	0	0	0		Ourworldindata
Iceland	76558	6493	1	85	0	0	0	0	0	0		Ourworldindata

India			1749417978		1232699				54933	363930701	73838055	VACCINATE INDIA[20]
Ireland	9798974	1786892	1218957	241728	0	0	0	0	1007	0		Ourworldindata
Italy	96513783	34368428	12174566	1508525	0	0	0	0	43329	0		Ourworldindata
Japan	299911417	83402847	117892	0	0	0	0	0	315582	0		Ourworldindata
Kenya	10801260		13675940	7414950	75000		300000					Africa CDC
Latvia	1624384	715409	262043	293843	0	9	27	0	438	0		Ourworldindata
Lesotho	696150		456000	2070580			203000					Africa CDC
Liberia	1417620		342000	3591444								Africa CDC
Libya	1665270		1730400	100000	1461250	150000	2000000					Africa CDC
Liechtenstein	22501	48607	0	264	0	0	0	0	1	0		Ourworldindata
Lithuania	3365494	329548	536498	295921	0	0	0	0	0	0		Ourworldindata
Luxembourg	746278	342272	105054	41510	0	0	0	0	498	0		Ourworldindata
Madagascar	7652970		794990	7209950								Africa CDC
Malawi	2093760		2567720	6269170								Africa CDC
Mali	1171260		597600	3333550		2735200	600000					Africa CDC
Malta	747236	279006	227875	32421	0	0	0	0	0	0		Ourworldindata
Mauritania	771080		2645350	2484000			1738000					Africa CDC
Mauritius	387270		500800	439200	250000	4000	1605000			200000		Africa CDC
Morocco	4956980		8723200	302400			46814000					Africa CDC
Mozambique	9788220		20446220	8989700			9723278					Africa CDC
Namibia	432900		526600	676800		100000	350000					Africa CDC
Nepal	1797582	7336243	14088349	3693423	0	0	19972478	0	0	0		Ourworldindata
Netherlands	24064293	11347843	2439668	750747	0	0	0	0	4097	0		Ourworldindata
Niger	640260		1196200	7939200		200000	1328800					Africa CDC
Nigeria	26394660		20741840	77927850			470000					Africa CDC
Norway	9935880	2404849	148207	7435	0	0	0	0	0	0		Ourworldindata
Peru	54422865	7463666	8187830	0	0	0	21338219	0	0	0		Ourworldindata
Poland	42007652	3847378	5292338	2731470	0	0	0	0	15868	0		Ourworldindata

Portugal	19036831	3938270	2302261	1141250	0	10285	5039	0	280	168			Ourworldindata
Romania	12914439	1008835	849559	2054653	0	0	0	0	0	0			Ourworldindata
Rwanda	14456600		7428830	1234860	227900		1345600						Africa CDC
Sao Tome and Principe	5850		153200	100800			100000						Africa CDC
Senegal	1364300		1979260	2569500	10000		1609318						Africa CDC
Seychelles	139000		1000		131120								Africa CDC
Sierra Leone	1308000	4491360	10000	200000	440000								Africa CDC
Slovakia	5195649	681585	844289	186499	37993	0	0	0	3740	0			Ourworldindata
Slovenia	2300847	235917	321165	135358	0	0	0	0	233	0			Ourworldindata
Somalia	1870560	9233300		290400	931600								Africa CDC
South Africa	28684331	0	0	9366280	0	0	0	0	0	0			Africa CDC
South Korea	52208403	13428975	20035346	1516759	0	0	0	0	263985	0			Ourworldindata
South Sudan	298520	5982170											Africa CDC
Spain	76812228	24379160	9796185	1982248	0	0	0	0	0	0			Ourworldindata
Sudan	4520960	20624300			1567600								Africa CDC
Sweden	17190312	4150890	1314000	0	0	0	0	0	7060	0			Ourworldindata
Switzerland	6215873	10641704	0	63651	0	0	0	0	3701	0			Ourworldindata
Tanzania	1065600	35226150		1000000	6578400								Africa CDC
Togo	919160	2620800		1638200	211200								Africa CDC
Tunisia	4286000	2540800	50000	3650000	150000							100000	Africa CDC
Uganda	6624480	22547800		6000000	2060400								Africa CDC
Ukraine	14774013	3044899	4041487	20680	0	9802231	0	0	0	0			Ourworldindata
United Kingdom	6288107	1041950	966494	1073633									Ourworldindata
United States	401685954	251852502	0	18991177	0	0	0	0	83047	0			Ourworldindata
Uruguay	2577190	0	92168	0	0	3249551	0	0	0	0			Ourworldindata
Venezuela*					0.23	0.064					0.62		PAHO
Zambia	1549300	12367050	50000		1995200								Africa CDC
Zimbabwe			65000	9700000	12632800					35000			Africa CDC

* Specific vaccination figures are not available for these countries; however, data on the usage proportions of various vaccines within each country is accessible.

S2 Estimates of administration costs of COVID-19 vaccines in each country

The administration costs of COVID-19 vaccines were firstly obtained from the Immunization Delivery Cost Catalogue (IDCC), a comprehensive dataset that consolidates unit costs of delivering vaccine for low- and middle-income countries. It has reviewed over 22,000 published papers from January 2005 to December 2023 and extracted the administration costs from these literatures. The study prioritized the incremental financial costs other than vaccine purchases in the IDCC dataset selected as the administrative cost. Then, a literature review was conducted to collect and organize the literature in accordance with the IDCC to obtain more information on the administrative cost in different countries. Data on the administrative cost per dose were obtained for 23 countries from the IDCC dataset and 46 countries from the literature review.

In cases where vaccination cost data were not available for a specific country by either of these methods, we applied a dual restriction based on income level and region subgroups. The costs of administration in countries without data were estimated by averaging the perceived costs in other countries within the same income level and region groups. If data on average administrative costs are absent in countries with both same income and regional dimensions, the average administrative costs of countries within the same income level would be used. For countries lacking classification by income level, such as Cape Verde, Vietnam, and Venezuela, the administrative costs were estimated based on geographic characteristics.

S3 Systematic Review

Nine databases have been searched, including PubMed, Cochrane Library, Embase, Scopus, Web of Science, Econlit, CNKI, Wanfang Data, CQVIP, for studies published by 1 June 2024. The search terms included (COVID-19 OR SARS-COV-2 OR Novel coronavirus) AND (vaccination OR immunization OR vaccine) AND (Cost OR Expense OR Expenditure OR Economic Evaluation OR Cost Minimization OR Cost-Effectiveness OR Cost-Benefit OR

Cost-Utility).

To obtain the administrative cost per dose of COVID-19 vaccine in different countries, attention was given to literature directly related to COVID-19 vaccines and studies employing economic methodologies, including cost estimation, cost-minimization analysis (CMA), cost-effectiveness analysis (CEA), cost-benefit analysis (CBA), and cost-utility analysis (CUA). The exclusion criteria included (1) literature for which full texts was not available; (2) non-original research, such as review articles, conference abstracts, or other publication types; (3) studies that were not evaluated from an economic perspective or lacked cost estimates; (4) literature focusing solely on the research, development, production, or marketing phases of COVID-19 vaccines, excluding costs associated with the vaccination process; (5) duplicate literature, with preference given to the most comprehensive articles; (6) literature not published in Chinese or English.

Initially, 14,687 articles were retrieved from nine databases. After removing 4,786 duplicates, 9,919 articles remained. Then a preliminary screening of these papers was conducted by with a review of their titles and abstracts by two reviewers, according to the exclusion criteria mentioned above. This process narrowed down the selection to 202 articles. Upon further in-depth reading of these 202 articles, we excluded 174 that did not meet the objectives of this study. Any disagreements during the screening process were resolved by a third reviewer. Ultimately, 28 articles were included in the analysis. The detailed screening process is illustrated in Figure S1 and Table S3.

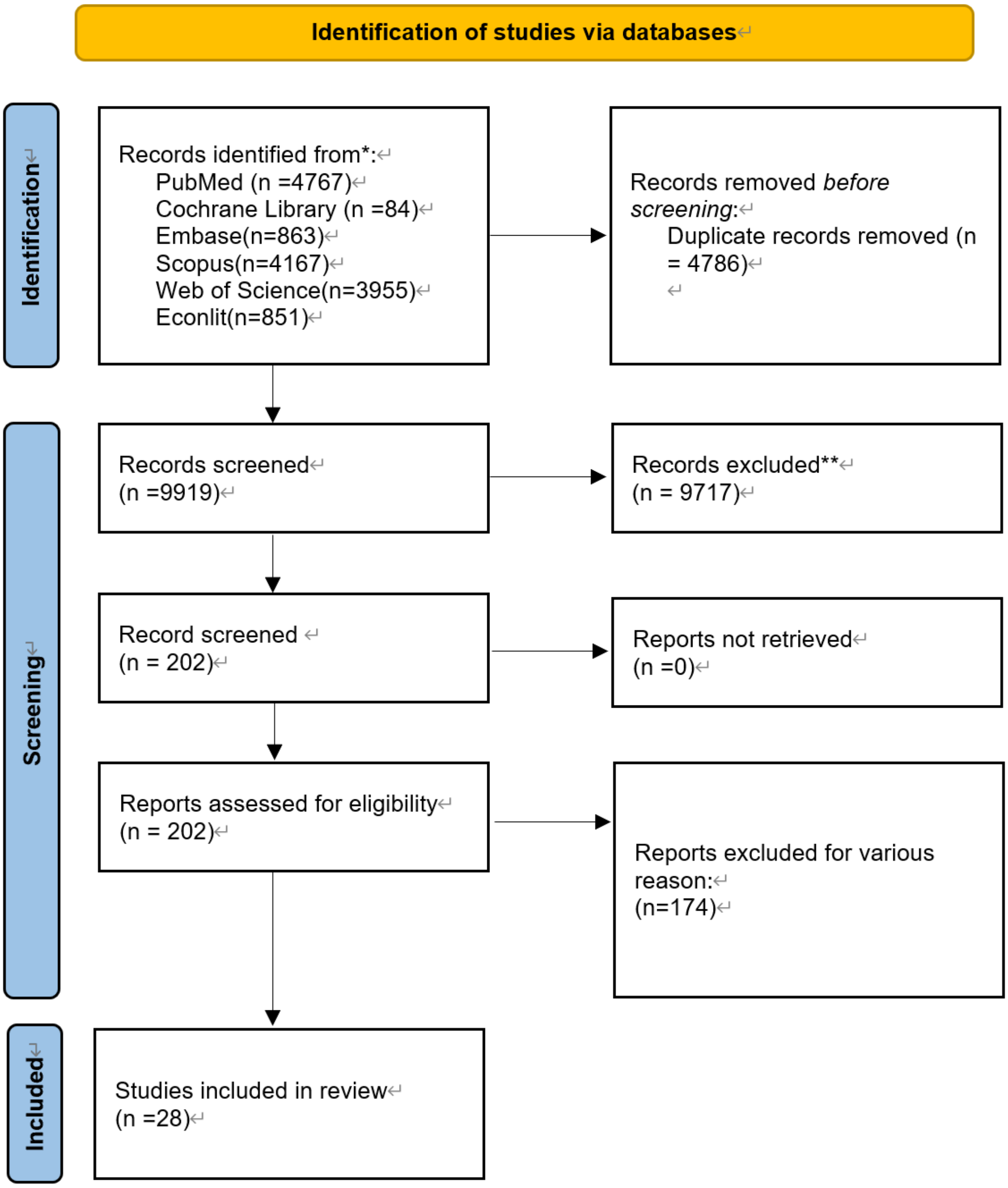

Figure S1 Flow Diagram for Literature Review

Table S3 Number of Records for 9 Databases

Database	Number of Records
PubMed	4767
Cochrane Library	84
Embase	863
Scopus	4167
Web of Science	3955
Econlit	851
CNKI	0
Wanfang Data	0

CQVIP	0
Total	14687

Reference

1. Meredith, S. Why Cuba's extraordinary Covid vaccine success could provide the best hope for low-income countries. Available online: **https://www.cnbc.com/2022/01/13/why-cubas-extraordinary-covid-vaccine-success-could-provide-the-best-hope-for-the-global-south.html** (accessed on 10/01).
2. World Health, O. *MI4A vaccine purchase data for countries*; World Health Organization: Geneva, 2019 2019.
3. PTI. Biological E reduces price of COVID-19 vaccine Corbevax to Rs 250. Available online: https://www.thenewsminute.com/news/biological-e-reduces-price-covid-19-vaccine-corbevax-rs-250-164018 (accessed on 10/01).
4. Bora, S. Here's The List Of Prices Of All COVID-19 Vaccines Authorised Around The World. Available online: https://www.shethepeople.tv/coronavirus/covid-vaccine-prices-india-and-the-world/ (accessed on 10/01).
5. Drugs. Novavax COVID-19 Vaccine Prices, Coupons, Copay Cards & Patient Assistance. Available online: https://www.drugs.com/price-guide/novavax-covid-19-vaccine (accessed on 10/01).
6. BMJ. What do we know about China's covid-19 vaccines? Available online: https://www.bmj.com/content/373/bmj.n912/rr-0 (accessed on 10/01).
7. Matin, H.; Saeed, K.M.I.; Hakim, M.S.; Naeemi, S. COVID-19 Vaccines Coverage in Afghanistan: a descriptive analysis of secondary data from DHIS2. *Razi International Medical Journal* **2023**, *3*, 62-69, doi:10.56101/rimj.v3i2.95.
8. Reuters. Albania gets 192,000 doses of Chinese Sinovac vaccine. Available online: https://www.reuters.com/article/us-health-coronavirus-albania-vaccines-idUSKBN2BH2N9/ (accessed on 10/01).
9. Albanian Goverenment Council of Ministers. 500,000 Pfizer's COVID vaccine doses secured. Available online: https://kryeministria.al/en/newsroom/sigurohen-500-mije-doza-te-vaksines-pfizer/ (accessed on 10/01).
10. Albanian Daily News. 50000 russian vaccine doses to come to albania next week. Available online: https://albaniandailynews.com/news/50-000-russian-vaccine-doses-to-come-to-albania-next-week (accessed on 10/01).
11. Agjencia Telegrafike Shqiptare. Mbërrin në Rinas partia e parë e 22 230 dozave Pfizer, që do të lëvrohen deri në 20 prill. Available online: https://ata.gov.al/2021/04/05/mberrin-ne-rinas-partia-e-pare-e-22-230-dozave-pfizer-qe-do-te-levrohen-deri-ne-20-prill/ (accessed on 10/01).
12. ABCNEWS. Albania receives 10,000 doses of russian Vaccine "Sputnik V". Available online: https://abcnews.al/albania-receives-10000-doses-of-russian-vaccine-sputnik-v

/ (accessed on 10/01).
13. A2NEWS. Albania receives 145,000 Pfizer vaccines, Rama: Politicians and analysts should not jump to conclusions. Available online: https://web.archive.org/web/20210420144557/https://english.a2news.com/2021/04/20/albania-receives-145000-pfizer-vaccines-rama-politicians-and-analysts-should-not-jump-to-conclusions/ (accessed on 10/01).
14. A2NEWS. Albania received 208,000 new doses of COVID-19 vaccines. Available online: https://web.archive.org/web/20210419171328/https://english.a2news.com/2021/04/19/albania-received-208000-new-doses-of-covid-19-vaccines/ (accessed on 10/01).
15. Inga Ting, K.S., Alex Palmer. Tracking Australia's COVID vaccine rollout numbers. Available online: https://www.abc.net.au/news/2021-03-02/charting-australias-covid-vaccine-rollout/13197518 (accessed on 10/01).
16. Worthington, B. Australia secures additional Pfizer vaccine following AstraZeneca concerns. Available online: https://www.abc.net.au/news/2021-04-09/national-cabinet-astrazeneca-covid-vaccine-clots-rollout/100058440 (accessed on 10/01).
17. 9NEWS. Australian government secures 85 million Pfizer booster shots for 2022 and 2023. Available online: https://www.9news.com.au/national/coronavirus-vaccine-rollout-85-million-pfizer-booster-doses-to-arrive-february-2022/2ec88b59-aa76-4a26-a1cb-4bdf997888ea (accessed on 10/01).
18. 9NEWS. Second shipment of Pfizer COVID-19 arrives in Australia, boosting national supply. Available online: https://www.9news.com.au/national/coronavirus-vaccine-second-pfizer-shipment-arrives-in-australia-boosting-rollout/b9f60f8e-7eed-4e1d-ae70-b96478fc7352 (accessed on 10/01).
19. Nozaki, I.; Hachiya, M.; Ikeda, C. COVID-19 vaccination program in Cambodia: Achievements and remaining challenges. *Glob Health Med* **2023**, *5*, 92-98, doi:10.35772/ghm.2023.01002.
20. VACCINATE INDIA. VACCINATE INDIA DASHBOARD. Available online: https://vaccinate-india.in/dashboard (accessed on 10/01).
